# Traditional knowledge of medicinal mushrooms and lichens of Yuman peoples in Northern Mexico

**DOI:** 10.1186/s13002-022-00550-8

**Published:** 2022-07-30

**Authors:** Joshua Anthuan Bautista-González, Adriana Montoya, Robert Bye, Martín Esqueda, María de los Angeles Herrera-Campos

**Affiliations:** 1 Departamento de Botánica, Instituto de Biología, Universidad Nacional Autónoma de México, Apdo. Postal 70-367, C.P. 04510 Cd. de México, México; 2 Posgrado en Ciencias Biológicas, Universidad Nacional Autónoma de México, Unidad de Posgrado, Edificio A, 1° Piso, Circuito de Posgrados, Ciudad Universitaria, Coyoacán, C.P. 04510, Ciudad de México, México; 3 Centro de Investigaciones en Ciencias Biológicas, Universidad Autónoma de Tlaxcala, Km 10.5 Autopista San Martín Texmelucan-Tlaxcala, 90120 Ixtacuixtla, Tlaxcala México; 4 Jardín Botánico, Instituto de Biología, Universidad Nacional Autónoma de México, Coyoacán, C.P. 04510, Ciudad de México, México; 5 Centro de Investigación Alimentación y Desarrollo A.C., C.P. 1735, 83000 Hermosillo, Sonora México

**Keywords:** Ethnomycology, Ethnolichenology, Traditional medicine, Yuman, Kiliwa, Paipai, Kumeyaay, Cucapá

## Abstract

**Background:**

Mushrooms and lichens are natural therapeutic resources whose millenary importance persists in indigenous and mestizo communities of Mexico. However, in this regard, in the northern part of the country there are few ethnobiological explorations. This study investigates the local knowledge of medicinal mushrooms and lichens used by Yuman peoples, whose native speakers are in imminent danger of extinction along with their biocultural heritage due to changes in their traditional primary activities and the usurpation of their ancestral lands.

**Methods:**

Ethnographic techniques in the field and standard lichenological and mycological methods in the laboratory were used.

**Results:**

Information was obtained on the medicinal use of 20 species, of which six are lichens of the genus *Xanthoparmelia* and 14 are non-lichenized fungi, mainly gasteroids. The latter are primarily used to treat skin lesions, while lichens are used in heart, urinary, and gastrointestinal diseases. The transmission of this local knowledge to future generations is discussed, as well as the intercultural cognitive convergence about the uses of medicinal mushrooms and lichens.

**Conclusions:**

The Yuman peoples preserve knowledge, practices and beliefs around mushrooms and lichens. Although increasingly less used, they still form part of their culinary and traditional medicine; even some are also used as ludic and ornamental purposes, and as trail markers. Beyond the pragmatic importance of these organisms, traditional knowledge about them is an essential part of the cultural identity that the Yuman peoples strive to preserve.

**Supplementary Information:**

The online version contains supplementary material available at 10.1186/s13002-022-00550-8.

## Background

For millennia, medicinal mushrooms and lichens have been recorded in Vedic, Greek, Egyptian, and Chinese cultures [1–5]. In Mexico, for centuries, the therapeutic use of sacred mushrooms has also been recorded [6]. In the sixteenth century, Sahagún reported the use of *teonanácatl*, a term meaning “divine mushroom” or “God’s flesh” in Nahuatl, corresponding to the genus *Psilocybe* [7]. In addition to its psychoactive properties, Sahagún described that it alleviated cold fever and gout [6, 8], he warned the natives that eating these mushrooms was prohibited because they were related to the devil [7]. Other mushrooms appear in the Florentine Codex with edible and therapeutic value such as *tzontecomananacatl*, *xelhuaznanacatl*, *chilmalnanacatl*, *menanacatl*, and *cacananacatl* [6, 8], although these have not been determined to species. This conception of mushrooms as food and medicine continues up to date in some indigenous groups of Mexico [9].

The application of lichens in traditional Mexican therapies has also been documented since the colonial period. Hernández [10] reported the use of lichens and recorded their Nahuatl names; among these, he mentions *ichcacalótic* or *tlapanquipatli* (“broken medicine”), which he describes as a foreign species of lichen, edible with a sweet taste, useful to extinguish fevers and resolves tumors. He also mentions the *xicauicalizpatli* that, in combination with several plants, was used to treat fever and may belong to the “genera of lichen or pulmonaria” [11]. However, despite the historical relevance of such records, the taxonomic identity of these lichens is unknown.

Currently, mushrooms and lichens are still present in the traditional medicine of various cultures of Mexico [12]. Until 2014, 202 species used in traditional Mexican medicine had been documented [9]. This number has increased to 330 after recent studies. Jiménez-Zárate reports 33 medicinal taxa in San Luis Potosí (unpublished observations) and Bautista-González [13] describes 115 medicinal lichens in Tehuacán-Cuicatlán region.

Mexico is one of the culturally richest countries about mushrooms, with more research, teaching, and diffusion on the topic [14]; however, still there are few ethnomycological studies focused on the knowledge of medicinal mushrooms and lichens, and even less in the northern part of the country. Among the medicinal mushrooms, *Bovista plumbea* Pers. has been recorded to treat skin lesions in Kumeyaay communities in Baja California [15] and *Podaxis pistillaris* (L.) Fr. to heal burns and cuts in Seri communities in Sonora [16]. Regarding lichens, *Usnea subfloridana* Stirt. was documented as used by Raramuri in Chihuahua to heal heart discomfort and other affections [17].

Northern Mexico is still inhabited by indigenous peoples whose languages are at risk of disappearing, such as the Yuman group [18]. Therefore, it is urgent to conduct ethnomycological and ethnolichenological research to contribute to the conservation of their culture and biocultural resources. The main aim of this study is to record the knowledge and use of mushrooms and lichens in the traditional medicine of Yuman communities in Baja California; peoples who are semi-nomadic belonging to the Yuman-Cochimi linguistic family. They inhabited all the ecosystems from the southwestern United States to the central desert of Baja California since the late prehistoric period (2500 BC), being the only survivor prehistoric group that established contact with the European, Mexican, and American colonizers [19]. Their linguistic family is composed of thirteen languages divided into four groups: (1) Kiliwa, (2) River (Mojave, Yuma, and Maricopa), (3) Delta-Californian (Cocopah, Tipai, Kumeyaay, and Diegueño), and (4) Pai (Yavapai, Walapai, Havasupai, Paipai, and Ku'ahl) [20].

In the early days, Yuman were primarily hunters and gatherers, but today they are sedentary, mainly dedicated to agriculture, livestock, collection, and commercialization of wild plants and handicrafts. These cultures are binational, dissociated after the dispossession of the Mexican territory by the United States. Moreover, since colonial times, they have been constantly attacked by hegemonic societies which have tried to exterminate them, harassed them for their lifestyle, and deprived them of their land. Now they have been confined to small territories that they themselves call “reservas” which are threatened by powerful national and transnational companies [21].

The communities visited in this study were found in critical situations. They lack or have inadequate basic services such as water, electricity, transportation, medical services, and schools. Unemployment has become one of the main reasons forcing Yuman people to emigrate, while drug trafficking and abuse have become the greatest problems for those who stay. The situation has turned so critical that, according to different media, the Kiliwa people signed a death pact to extinguish themselves as an ethnic group, they agreed not to reproduce anymore due to historical injustices and the government's abandonment [22].

This research is the first study focused on preserving the knowledge of mushrooms and lichens used as medicine among the Yuman groups from Northern Mexico.

## Methods

### Study area

**Fig. 1:**
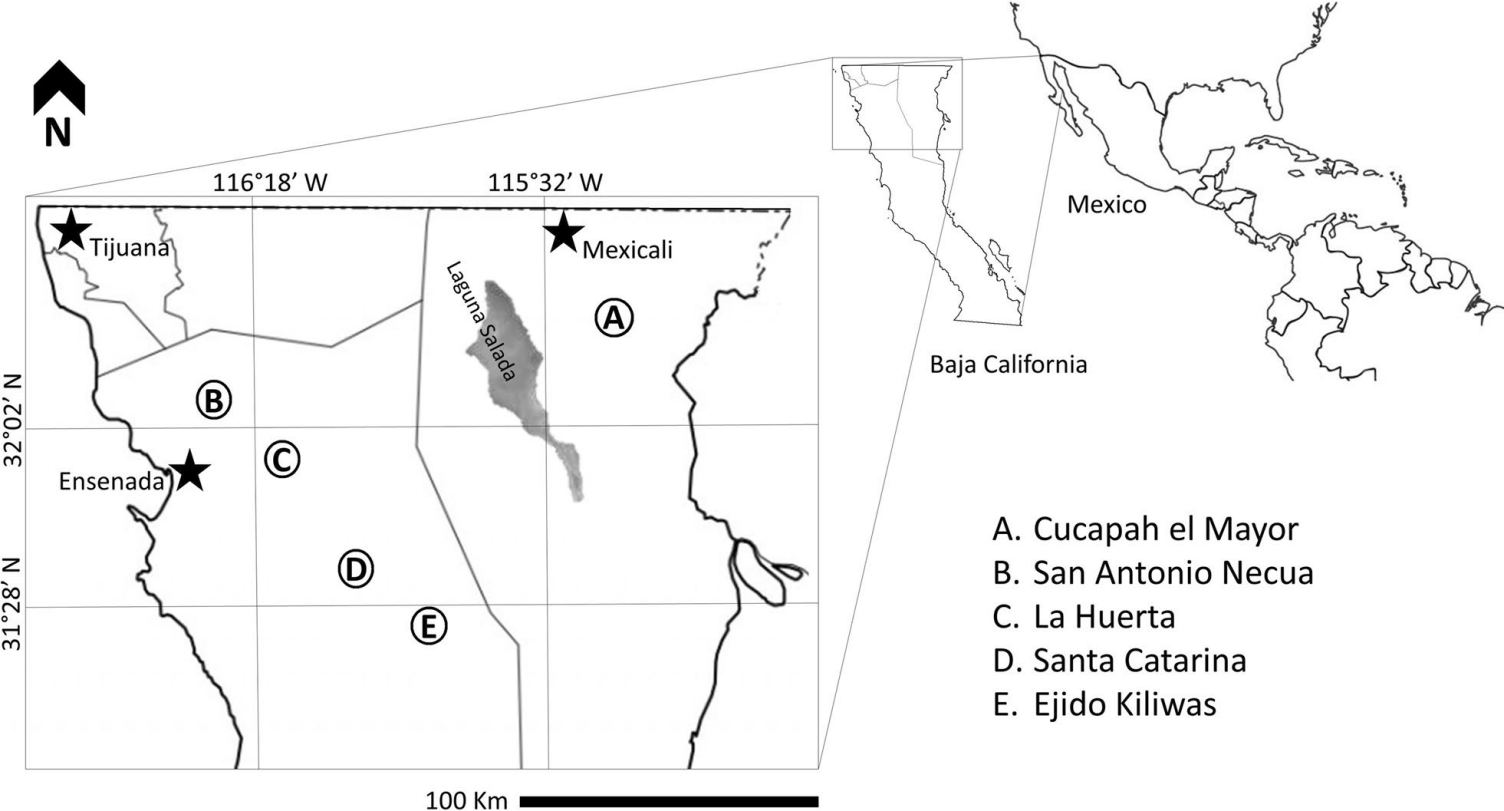
Study sites

The five communities of Baja California considered in this study: Cucapah el Mayor, Ejido Kiliwas, Santa Catarina, La Huerta y San Antonio Necua (Fig. 1), all associated with dry (B) and semi-arid (BS) climates, xerophilous scrub as dominant vegetation, and riparian forests with *Platanus racemosa* Nutt. ex Audubon, *Populus tremuloides* Michx., *P. freemonti* S. Watson, *P. trichocarpa* Torr. & A. Gray ex Hook., *Quercus agrifolia* Neé, and *Salix* spp. [23]. Cactaceae and *Yucca* spp. are present in all localities, besides small patches of *Pinus* in La Huerta and *Quercus* in San Antonio Necua. Except for Cucapah el Mayor, located between the Colorado Riverbank and the foothills of the Sierra Cucapá, all the communities are in Ensenada municipality, in the Mediterranean area in the interior of the Baja California peninsula [24].

### Population

Cucapah el Mayor is one of the three Cucapá communities that persist, together with another in Arizona, USA, and one more in Sonora, Mexico [19]. Cucapá means “*river people.*” In this community, only two persons still speak Cocopah language, and another three locals only know some words.

Ejido Kiliwas is the only locality Kiliwa that still exists today. They are recognized as “*sierreños*” (highlanders) because the places of their ancestors are located near San Pedro Mártir [19]. Kiliwa means “*our people*” or “*the hunters.*” Only four native speakers remained at the time of the first visit; unfortunately, Mrs. Hipólita Espinoza, who strongly promoted her culture, passed away before the second visit.

La Huerta and San Antonio Necua are Kumeyaay communities, part of a binational ethnic group settled in Baja California, Mexico, and California, USA [19]. Among Yuman languages, Kumeyaay has the highest number of speakers; according to this study, there were 14 speakers found in both communities. Kumeyaay means “*people who live in the mountains,*” “*ravine people,*” or “*people from the coast.*”

Santa Catarina is a Paipai community, even though some people speak Ku’ahl, and few speak both languages. They are also known as "*sierreños*" (highlanders) for being in the vicinity of the Sierra Juárez [19]. Paipai means “*living people.*” It should be noted that the information on the number of speakers per locality and the meaning of the cultures were obtained according to data provided by traditional authorities and field observations. Since the official documents do not agree with the reality observed in the field. For example, the 2010 census does not record native Ku'ahl speakers [25], but we interviewed three of them.

### Field work

Before the fieldwork, permission and collaboration were requested from local and municipal authorities through a written letter, as well as orally, and following the recommendations of the ISE and SOLAE [26, 27].

Sixty-three semi-structured individual or collective interviews were conducted with 71 seventy-one connoisseurs (Table 1); in six two or three people took part. The snowball technique [28] was used to contact people recognized by the members of their own locality as wise, native speakers, and traditional doctors, and to find older adults. The free listing technique was applied at the beginning of the interview [29]. Photographic and organic stimuli of mushrooms and lichens (foliose, fruticose, crustose) collected in the region were shown.

**Table 1:** Inhabitants by locality and speakers of Yuman languages

Locality	Population	Language	Native speakers	Number of interviewees
Cucapah el Mayor	173	Cucapá	2	17
Ejido Kiliwas	13	Kiliwa	3	10
Santa Catarina	133	Paipai	5	12
		Ku’ahl	3	
La Huerta	131	Kumiai	6	9
San Antonio Necua	204	Kumiai	8	12

All interviewed people speak Spanish

During interviews we investigated local knowledge about medicinal mushrooms and lichens (local nomenclature, classification, habitat, morphology, substrate, and ecology) and their medicinal use (illnesses, used part, preparation formula, and way of administration).

In addition to the mentioned localities, three interviews were conducted in Ejido Puerta Trampa, near La Huerta, where some residents of the Kumeyaay and Yaqui cultures live. Two individuals from indigenous communities (La Huerta y Ejido Kiliwas) were also interviewed in Ensenada city, where they spend prolonged periods for health or work reasons.

Fifty-five percent of the 63 interviewees were women, 45.0% were men; 80.0% were over 50 years old; 55.0% were between 50 and 68, 13 were over 70, three 80, and one woman was 102 years old. Regarding young people, only a ten-year-old boy and a 26-year-old woman were interviewed; the remaining eleven were between 33 and 47 years old.

Studied specimens were collected during field trips guided by local connoisseurs. The special orders technique was also used; it consists of asking a person, if finding the mushroom or lichen, to save the specimen for a next visit [30].

Taxonomic determination of the specimens was carried out by observation of macro and microscopic characters as well as using specialized keys [31–35].

In the case of lichens, chemical analyses such as spot tests and thin layer chromatography were conducted. All the specimens were deposited in the National Herbarium of Mexico (MEXU) at UNAM.

## Results

One hundred sixty-one specimens were collected, corresponding to 65 taxa, of which 38 are used mainly as medicine, food, and/or ornament (Table 2). Nineteen interviewees reported 20 species used for therapeutic purposes, 14 non-lichenized fungi, mainly gasteroids, and six lichens of the genus *Xanthoparmelia.* The first ones are mainly used to treat skin conditions, while lichens are employed to treat heart and urinary tract diseases. In addition, the dietary use of eight fungal taxa and nine ornamental lichen species were documented.

**Table 2:** Mushrooms and lichens found in the Yuman territories in Baja California Norte, Mexico

Taxa	Local name, use and locality
*Non-lichenized fungi*	
Agaricoid sp. Bautista-González 1200219	*Jlhaush asaw* (e) (4)
*Agaricus campestris* *sensu* Cooke Bautista-González 1250918, 88100219, 10100219, 10110219, 54200219	*Mat jumiy* (*mat* = earth), *hong mat pach* (mushroom that born in the ground), hongo de tierra (soil fungus) or hongo rosado (pinkish mushroom) (e) (3)/hongo de tierra (u) (4)
*Amanita* sp. Bautista-González 20060219	*Kook lieb* (u) (2)
*Astraeus hygrometricus* (Pers.) Morgan Bautista-González 2230918	*Kak ñaup* (m) (4)
*Battarrea phalloides* (Dicks.) Pers. Bautista-González 88040219	*S'pooy ku´looy* (u) (1)
*Boletus* sp. (parasitized) Bautista-González 14110219	Hongo (u) (3)
*Chlorophyllum* sp. Bautista-González 8110219, 2110219	*Kaak ñusip* (m) (3)
*Clitocybe* sp. Bautista-González 4110219	Hongo (u) (3)
*Conocybe* sp. Bautista-González 54100219, 20180219	Hongo (u) (3 and 5)
*Coprinellus* sp. Bautista-González 31030219, 304102018, 51200219	*S'pooy m'pool* (little hat mushroom) or hongos conitos (small cone mushrooms) (e) (1)/*kook lieb* (u) (2)/blancos (white) (u) (4)
*Coprinus* sp. Bautista-González 11110219, 52200219	Hongo (u) (3)/Blancos (u) (4)
*Coriolopsis gallica* (Fr.) Ryvarden Bautista-González 16060219, 17060219, 18060219	*Meltaay s'pooy* (poplar mushroom) or *ijáau s'pooy* (willow mushroom) (u) (1)/*Iisac* o *iwuil saac* (fungus sticking from the tree) (*iwuil* = plant or branch; *saac* = leaves, because it is fan-shaped) in Paipai or hongo in Spanish (m) (2)
*Cyathus* sp. Bautista-González 3110219	Hongo (u) (3)
*Disciseda candida* (Schwein.) Lloyd Bautista-González 6110219	*Kaak ñusip* (m) (3)
*D. hyalothrix* (Cooke & Massee) Hollós Bautista-González 333020219d	*S'pooy ku´looy* (u) (1)/*kaak ñusip* (m) (3)
*Geastrum floriforme* Vittad. Bautista-González 12110219a	*Kaak ñusip* (m) (3)
*G. kotlabae* V.J. Staněk Bautista-González 14060219, 1110219, 7110219	Unregistered local name (u) (2)/*kaak ñusip* (m) (3)
*Itajahya galericulata* Möller Bautista-González 1200918	*S'pooy ku´looy* (u) (1)
*Inonotus cuticularis* (Bull.) P. Karst. Bautista-González 15200918, 2200918	*Meltaay s'pooy* (poplar mushroom) or *ijáau s'pooy* (willow mushroom) (e?) (1)
*Lepiota* sp. Bautista-González 204102018	*Kook lieb* (u) (2)
*Lycoperdon* cf. *candidum* Pers. Bautista-González 1230918	*Kak ñaup* (m) (4)
*Montagnea arenaria* (DC.) Zeller Bautista-González 20110219, 53200219	*S'pooy ku´looy* (u) (1)/hongo (u) (3)/negro (black) (u) (4)
*Omphalotus* sp. Bautista-González 46100219	Hongo (u) (3)
*Panaeolus* sp. Bautista-González 19060219	*Kook lieb* (u) (2)
*Peziza* sp. Bautista-González 55200219	Hongo café (brown fungus) (u) (4)
*Phellinus* sp. Bautista-González 30220918, 31220918, 49200219	*Meltaay s'pooy* (poplar mushroom) or *ijáau s'pooy* (willow mushroom) (e?) (1)/*jlhaush* (e?) (4)/hongo (e?) (5). They consider it edible when it is immature, but possibly they confuse it with another lignicolous species
*Pholiota* sp. Bautista-González 15020219	*S'pooy ku´looy* or hongo malo (u) (1)
*Pisolithus tinctorius* (Pers.) Coker & Couch Bautista-González 41100219, 42100219, 21180219, 50200219	*Kaak ñusip* (m) (3)/*kak ñaup* (m) (4)/hongo de polvo (powder fungus) (u) (5)
*Podaxis pistillaris* (L.) Fr Bautista-González 1290918, 1160219, 1170219	Hongo, hongo de polvo, hongo de polvo negro, hongo que echa polvo negro, hongo que suelta tinta negra (fungus that releases dust or black ink), hongo que echa polvo venenoso (poisonous dust), hongo bule, hongos chilos (chilo ≈ chido, cool, alluding to its alleged narcotic properties), hongo para fumar or hongo alucinante (smoking or hallucinatory mushroom) (m) (6)
*Stropharia* sp. Bautista-González 32100219, 33100219	Hongo (u) (3)
*Tricholoma equestre* (L.) P. Kumm. Bautista-González 43100219, 2200219	Hongo (u) (3)/hongo de encino (oak mushroom) (e) (4)
*T. portentosum* (Fr.) Quél. Bautista-González 44100219, 45100219	Hongo (u) (3)
*T. sejunctum* (Sowerby) Quél. Bautista-González 3200219, 4200219	Hongo del sauce or sauco (willow mushroom) (e) (4)
*Tulostoma fimbriatum* Fr. Bautista-González 1260918, 333020219a, 1030219, 15060219, 11100219, 9110219	*S'pooy ku´looy* (u) (1)/unregistered local name (u) (2)/*kaak ñusip* (m) (3)
*T. pygmaeum* Lloyd Bautista-González 383020219	*Kaak ñusip* (m) (3)
*Ustilago maydis* (DC.) Corda (not collected, identified by photography.)	Hongo de maíz or hongo de elote (corn or maize mushroom) (e, m) (1)/*kook tiech tulish* (*tiech* = corn; *tulish* = glued), *tiek mach*, hongo de elote or hongo de maíz (e) (2)/*chomat-michap* (u) (3)/hongo de elote (u) (4)/hongo de maíz (u) (5)/*jchas ñiígl* (*jchas* = maíz; *ñiígl* = louse), piojo del maíz, hongo de elote, hongo de maíz or hongo de maíz azul (e) (6)
*Volvariella bombycina* (Schaeff.) Singer Bautista-González 1220918, 104102018	*Kook, kook jan* (*jan* = good, well), *ja kichpach mab* (that which grows from the poplar is edible), hongo, hongo de álamo or hongo de tronco de álamo (poplar trunk fungus) (e) (2)/hongo de álamo (e) (4)
*Volvopluteus* sp. Bautista-González 47100219, 49100219, 5110219, 1070219, 19180219, 22180219	Hongo de tierra (soil fungus) (e) (3)/hongo or hongo, hongo de álamo (e, m) (5)
*Xerocomus* sp. Bautista-González 48100219	Hongo (u) (3)
*Xylaria* cf. *polymorpha* (Pers.) Grev. Bautista-González 1190918	Hongo (u) (7)
*Lichens*	
*Acarospora* spp. Bautista-González 91200918, 94200918, 95020219, 93200219	*Uja' tebiyauup* (*uja'* = stone; *tebiyauup* = flower) or flor de piedra (stone flower) (d) (1)/lama (≈slime) (u) (4)
*Caloplaca* sp. Bautista-González 93200918a, 102200918	*Uja' tebiyauup* or flor de piedra (d) (1)
*Candelariella* sp. Bautista-González 94020219a	
*Cladonia* cf. *subreticulata* Ahti Bautista-González 101230918	Musgo (moss) (u) (4)
*Heterodermia* cf. *commosa* (Eschw.) Follmann & Redón Bautista-González 91092018	Unregistered local name (d) (7)
*Lecanora* sp. Bautista-González 98200918	*Uja' tebiyauup* or flor de piedra (u) (1)
*Letharia columbiana* (Nutt.) J.W. Thomson Bautista-González 92230918, 93230918, 102230918	Flor de la manzanita or brote de la manzanita (manzanita’s bud) (d) (1)*/toji* de manzanita (d) (4)*/toji* (d) (7)
*Nephromopsis merrillii* (Du Rietz) Divakar, A. Crespo & Lumbsch Bautista-González 91230918	*Toji* (u) (4)
*Niebla* sp. Bautista-González 93092018	*Toji* (d) (7)
*Oxneria fallax* (Arnold) S.Y. Kondr. & Kärnefelt Bautista-González 95200918, 101200918a	*Ipa tebiyauup* (stick flower) (u) (1)
*Physcia stellaris* (L.) Nyl. Bautista-González 101200918b, 91250918, 94100219	*Ipa tebiyauup* (u) (1)/lama (u) (3)
*Protoparmeliopsis muralis* (Schreb.) M. Choisy Bautista-González 93200918b, 99200918, 94020219b	*Uja' tebiyauup* or flor de piedra (d) (1)
*Ramalina menziesii* Taylor Bautista-González 94092018	*Toji* (d) (7)
*Scytinium* sp. Bautista-González 94200219, 97230918b	Lama (u) (4)
*Teloschistes chrysophthalmus* (L.) Th. Bautista-González 92092018	Unregistered local name (d) (7)
*Umbillicaria* sp. Bautista-González 97030219	*Uja' tebiyauup* or flor de piedra (u) (1)
*Xanthoparmelia isidiigera* (Müll. Arg.) Elix & J. Johnst. Bautista-González 92030219	*Uja' tebiyauup* or flor de piedra (m) (1)
*X. joranadae* (T.H. Nash) Egan Bautista-González 92200918, 97200918	
*X. lineola* (E.C. Berry) Hale Bautista-González 93030219, 92220918, 94230918, 96230918, 97230918, 98230918, 100230918, 95041018, 91100219, 97041018, 93020219, 98020219, 91050219, 91060219, 92200219	*Uja' tebiyauup* or flor de piedra (m) (1)/*wui tabsh* or *wui mokual* (rock skin) (m) (2)/lama (u) (3)/*wui tabsh*, flor de piedra or zacatito (little grass) (m) (4)/flor de piedra (m) (5)
*X. maricopoensis* T.H. Nash & Elix Bautista-González 97020219	*Uja' tebiyauup* or flor de piedra (m) (1)
*X. mexicana* (Gyeln.) Hale Bautista-González 100200918, 91220918, 95230918, 99230918, 92041018, 93041018, 94041018, 96041018, 98041018, 99041018, 91020219, 92020219, 100020219, 102020219, 95030219, 92100219, 93100219, 91200219	*Uja' tebiyauup* or flor de piedra (stone flower) (m) (1)/*wui tabsh* or *wui mokual* (m) (2)/lama (u) (3)*/wui tabsh*, flor de piedra or zacatito (m) (4)/flor de piedra (m) (5)
*X. novomexicana* (Gyeln.) Hale Bautista-González 91030219, 91041018	*Uja' tebiyauup* or flor de piedra (m) (1)/*wui tabsh* or *wui mokual* (m) (2)
*Xanthoparmelia* sp. (not collected)	*Wui ñiígl* (*wui* = stone), piojo de piedra (stone louse), piojo del cerro (hill louse), moho (mildew), lama or lamita (u) (6)

Localities: (1) Ejido Kiliwas, (2) Santa Catarina, (3) San Antonio Necua, (4) La Huerta, (5) Ejido Puerta Trampa, (6) Cucapá El Mayor, (7) Ensenada. Uses: (m) medicinal, (e) edible, (d) decorative, (u) unused. Note: Yuman names are in italics, those in Spanish are shown without italics, and the meaning in English in parentheses. The sign "?" indicates taxa that were referred to as alimentary, but their edibility is uncertain

### Medicinal mushrooms

**Fig. 2:**
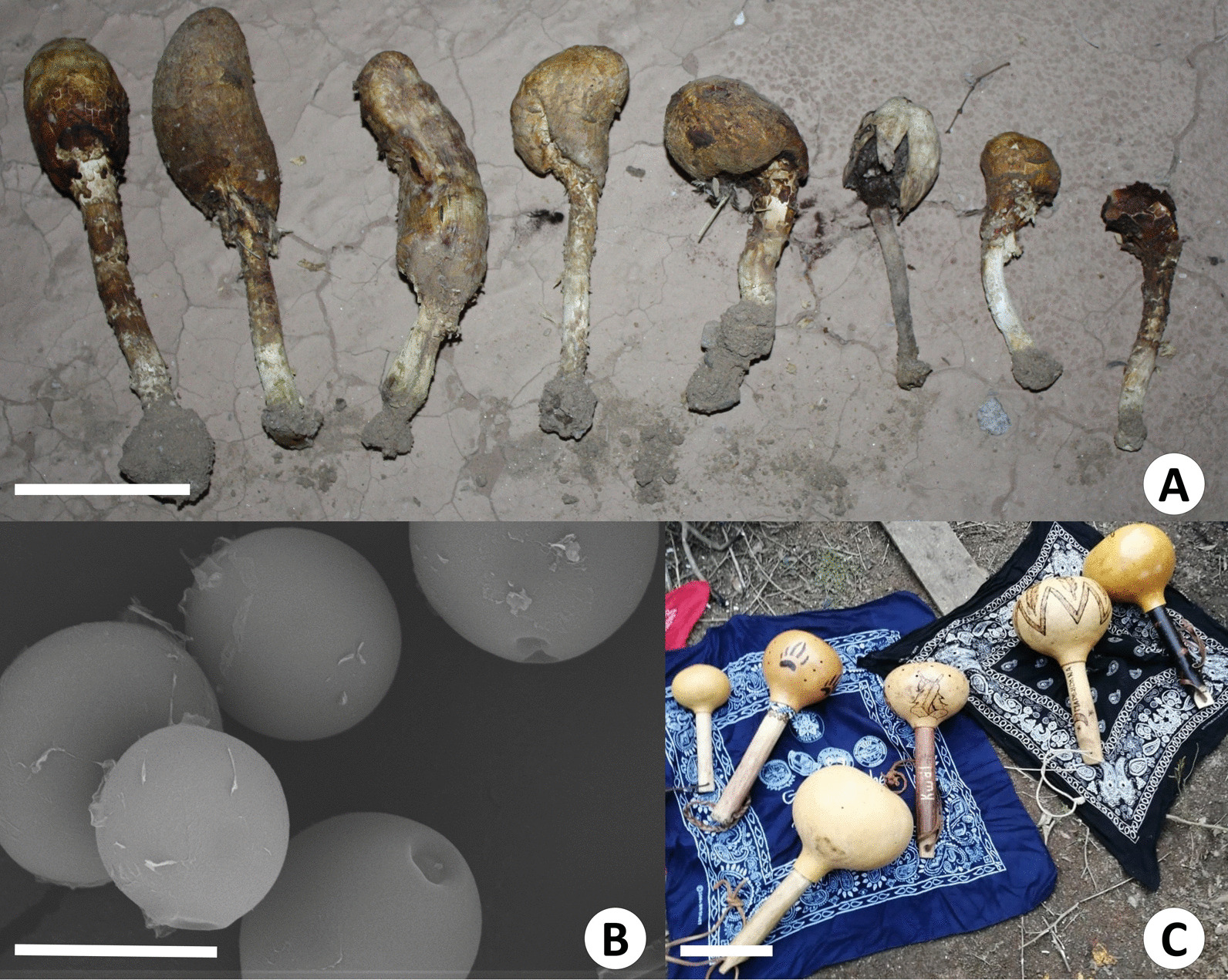
**A** *Hongo bule* (*Podaxis pistillaris*) in sandy soils in the vicinity of Cucapah el Mayor. **B** Smooth spores under SEM, image by D. Delgado. **C** *Bules* used as sound instruments (maracas) in Kumiai ceremonies, image by Anselmo Domínguez. Scales: A = 5 cm, B = 10 5 µm, C = 10 cm

In Cucapah el Mayor, eleven Spanish local names were documented to name *Podaxis pistillaris*, although its Cucapá denomination was not reported. It is known as *hongo de polvo negro* (black powder mushroom) because of the mature spore mass color; or *hongo bule* due to its resemblance to the *bule* (bottle gourd, *Lagenaria siceraria* [Molina] Standl.) used to make musical instruments (Fig. 2).

Three respondents use *P. pistillaris* to treat skin conditions, such as infected or not bleeding wounds, sores, cuts, scrapes, burns, and skin infections. They clean the skin and place the spores on the affected area to anesthetize, close the wound and stop the bleeding. One of them mentioned that the spores are also sprayed directly to the face to anesthetize the persons and make them sleep, but with the warning to wash the hands after handling this remedy since the mushroom can be harmful.

Kumeyaay peoples also appreciate gasteroid mushrooms for their medicinal attributes. In La Huerta, four interviewees recognize *Astraeus hygrometricus*, *Lycoperdon* cf. *candidum*, and *Pisolithus tinctorius* as medicine. Altogether they are called *kak ñaup* (crow’s tobacco) or *kak shit* (crow’s poop) because they expel “smoke” (spores), and crow because the mushrooms are dark, like these birds. The spores are used to treat skin (*mat*), injuries such as *akath* (bleeding wounds), minor scrapes and cuts, mutilations or wounds caused by firearms, burns, and infections. According to local experts, this remedy helps close and scar the wound, *juath spaawa* (stop bleeding). An interviewee recommends, before applying the spores, washing the wound with plants such as *moronel* (*Lonicera subspicata* Hook. & Arn.) and *golondrina* (*Euphorbia micromera* Boiss.).

**Fig. 3:**
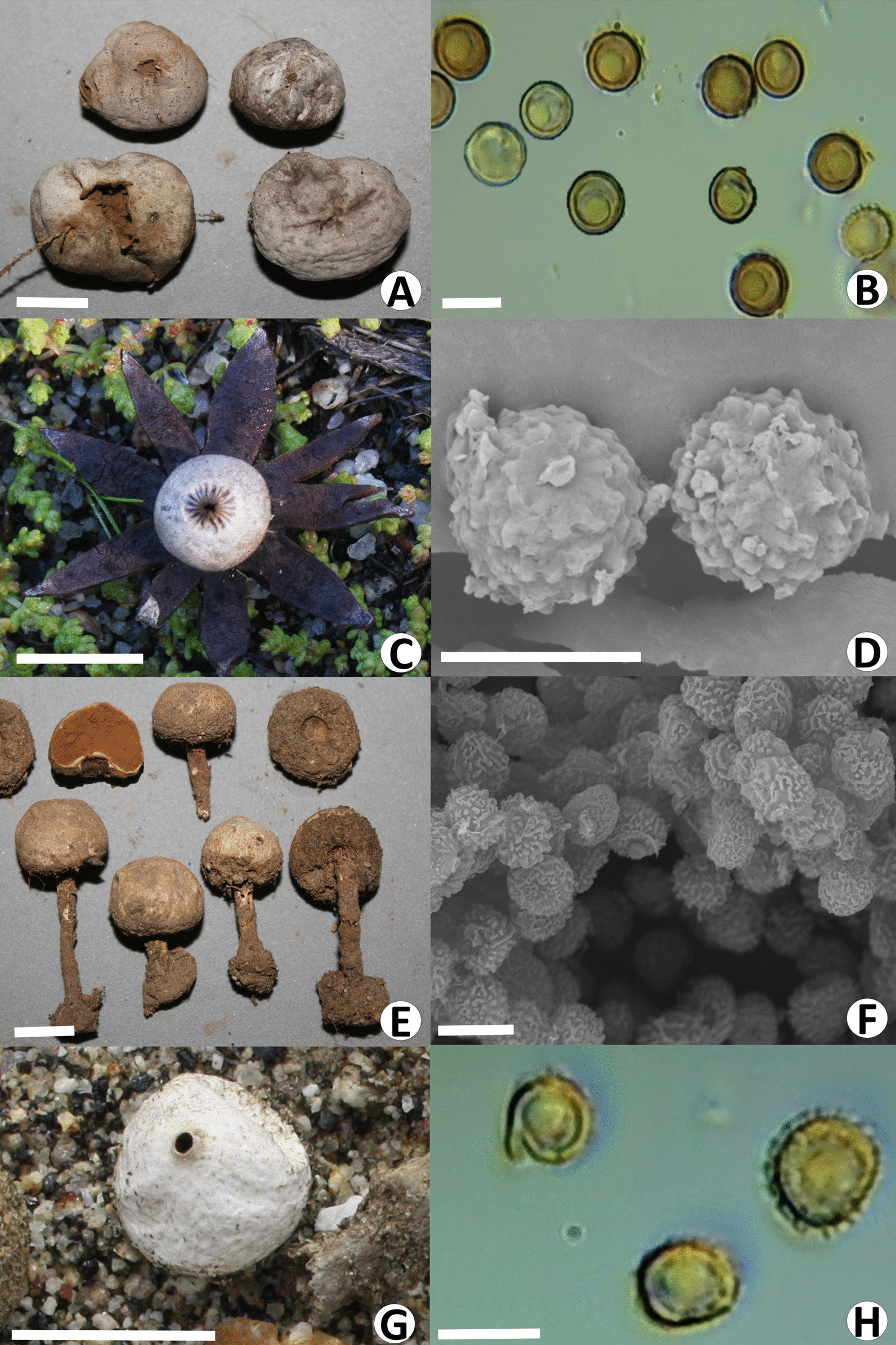
Some medicinal mushrooms of San Antonio Necua and their spores. **A**, **B** *Disciseda candida*, **C**, **D** *Geastrum kotlabae*, **E**, **F** *Tulostoma fimbriatum*, SEM image by D. Delgado. **G**, **H** *T. pygmaeum*. Scales: A, C, E and G = 1 cm; B, D, F and H = 5 µm

In San Antonio Necua (Kumeyaay community), four interviewees mention the medicinal use of eight gasteroids: *Chlorophyllum* sp., *Disciseda candida*, *D*. *hyalothrix*, *Geastrum floriforme*, *G*. *kotlabae*, *Pisolithus tinctorius*, *Tulostoma fimbriatum*, and *T*. *pygmaeum* (Fig. 3). These are not considered mushrooms in the Kumeyaay classification, so they did not appear in the free lists. Gasteroids are conceived and named as *kaak ñusip* (*kaak* = crow; *sip* = to smoke; *ñusip* = cigarette or tobacco), *kaak up*, *kaak ñu up* (*up* = cigarette), *cigarro del cuervo* (crow cigarette), or *tabaco de cuervo* (crow tobacco) because they have seen that these birds smoke the *humo* (smoke = spores). Also named *bolsitas para cortadas* (little bag for cuts) by a woman. These mushrooms are used to heal skin lesions: bleeding and surgical wounds, wounds, scrapes, cuts, sores, and burns. As well as for the *rasquera* (≈ itch), a disease characterized by dryness, redness, irritation, and intense itching caused by contact with chilly water, soil, or soap; by sunstroke, and because “*pega el Norte*” (cold front strikes). Local experts attribute astringent and hemostatic properties to these gasteroids. The interviewees also say these mushrooms are beneficial for facial skin to reduce wrinkles.

The spores, called *mat naj* (*tierrita*—little earth) or *polvito* (dusty), are applied directly to previously washed skin as plaster and left uncovered or covered with a cloth.

To relieve skin conditions with gasteroids, Cucapá and Kumeyaay repeat the treatment as many times as necessary, only once in case of minor wounds or several times a day for weeks in severe injuries.

**Fig. 4:**
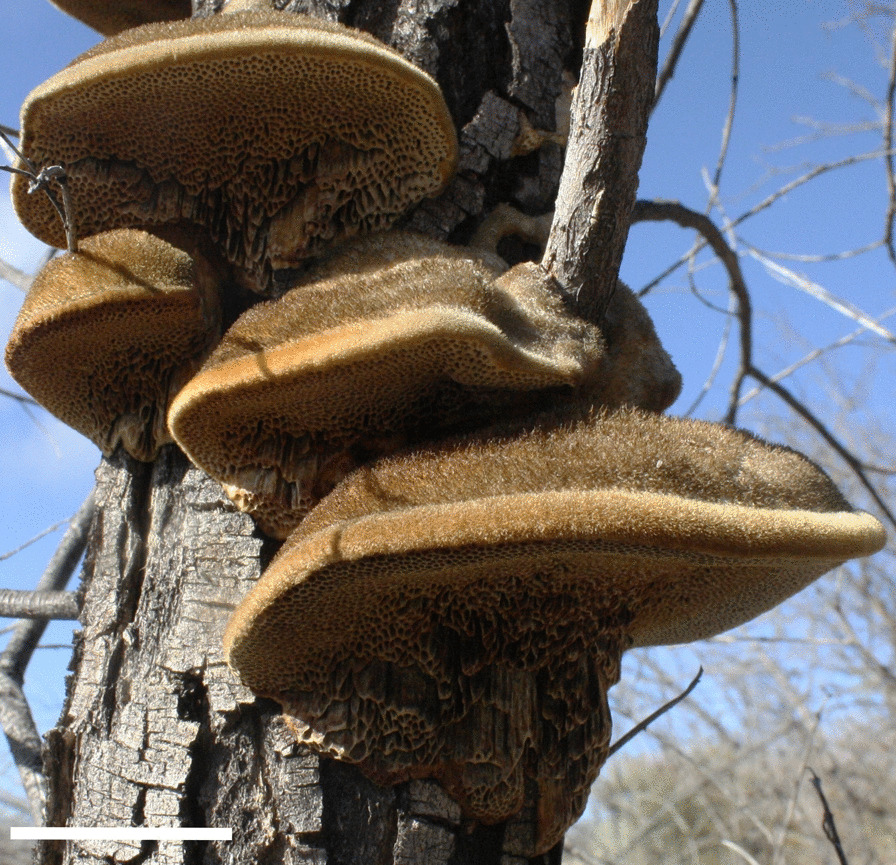
Medicinal polyporoid *Coriolopsis gallica* growing on a dead trunk. Scale = 5 cm

In Santa Catarina, a Paipai woman referred the use of *Coriolopsis gallica* (Fig. 4) to treat diabetes, a condition known as *muil sharau* (sugar disease). She explains that it affects the *shjoat* (blood), patients turn pale and sad; it is caused by excessive intake of soda or honey; she recommends avoiding sweets, butter, and flour during treatment. According to her, if diabetes is treated early with this mushroom, it can be cured, but at an advanced stage, it only controls it. The preparation consists of boiling an esporome per cup of water and drinking it as *agua de uso* or *agua de tiempo* (time water), meaning the intake of the infusion (hot or cold) throughout the day on a regular basis instead of water, up to several months.

### Medicinal lichens

**Fig. 5:**
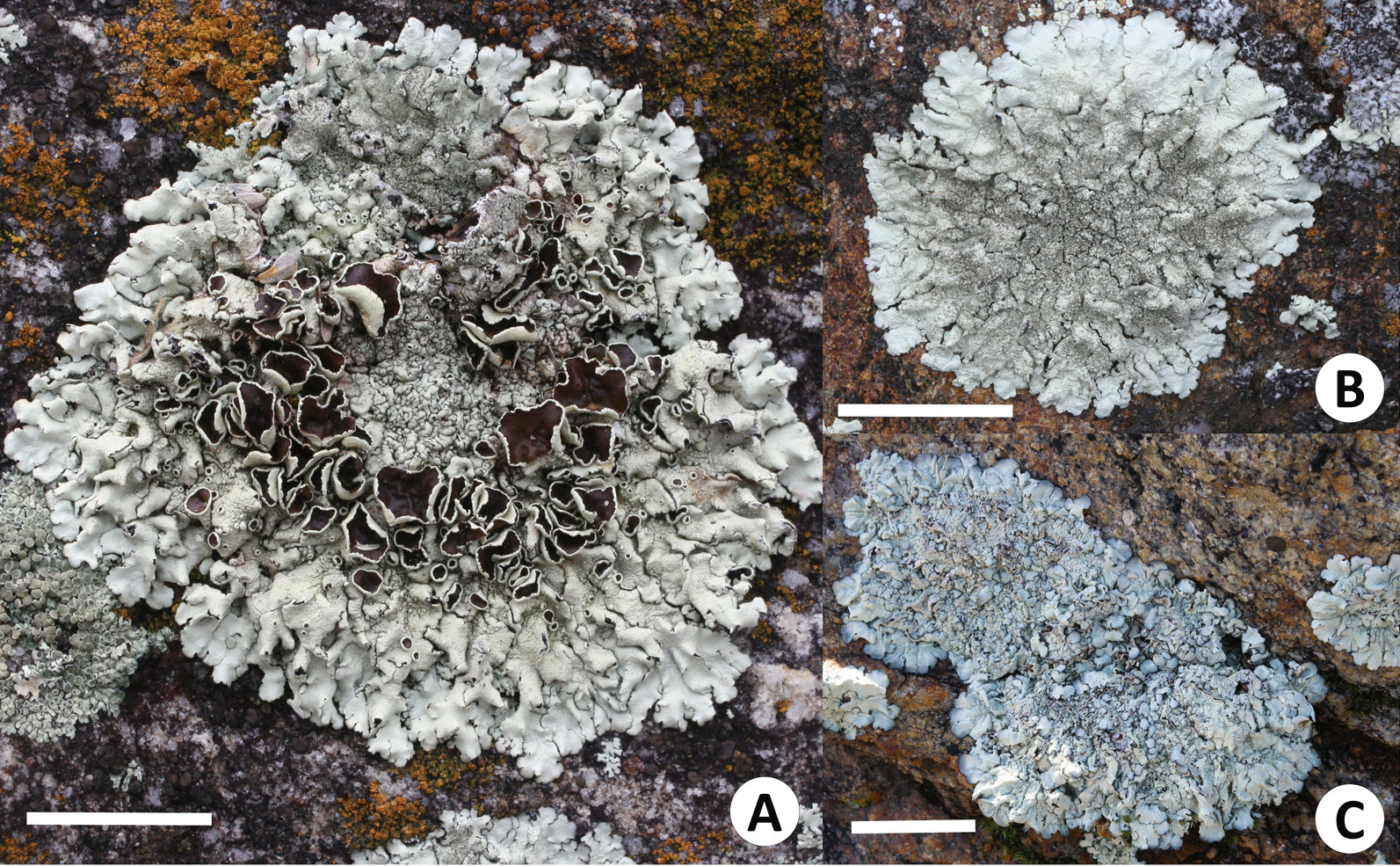
Medicinal lichens collected in Ejido Kiliwas. **A** *Xanthoparmelia lineola*. **B** *X. isidiigera*. **C** *X. joranadae*. Scales: A, B and C = 2 cm

The use of six medicinal lichens was reported by three interviewees from the Kiliwa community: *Xanthoparmelia isidiigera*, *X. joranadae*, *X. lineola*, *X. maricopoensis*, *X. mexicana*, and *X. novomexicana* (Fig. 5). Mrs. Leonor Farldow, a distinguished local connoisseur, calls *Xanthoparmelia* spp. as *uja' tebiyauup* (stone flower), and *ipa tebiyauup* (stick flower) to the foliose corticolous lichens and mentions that both are used as a remedy. All these lichens are used to relieve ailments of *sempaapo* (kidney), bladder, and urinary tract in general, conditions known as *sitam micha*, *sit jaam ju'looy* (*sit* = go out; *u'looy* = bad), or *mal de orín* (bad urine), whose recurrent symptoms are scanty urination with pain or burning, as well as bleeding in severe cases.

In the Kiliwa dictionary, *mal de orín* appears as *pamaay cheen* (*pamaay* = to urinate) [36]. According to the interviewees, this is caused by eating acidic foods such as immature fruits or walking barefoot. They attribute to the lichens a diuretic effect, bladder purifier, and renal healing. These are also employed to treat *lepée* (liver) diseases, such as cirrhosis; and to treat *kuti'p gap* (*kuti'p* = heart; *gap* = pain or illness) caused by anger, depression, or intense gratifying emotions. According to local perception, feelings turn into blood clots causing cardiac disturbances, and lichens can unclog the fat in the arteries.

In four interviews conducted in Santa Catarina, the traditional medicinal knowledge of *Xanthoparmelia lineola*, *X*. *mexicana*, and *X*. *novomexicana* was documented. The local names for saxicolous lichens are registered in Spanish, Ku’ahl, and Paipai. Spanish: *flor de piedra* (stone flower), *lama*, *alfombrita* (little carpet) or *hongo de piedra* (stone mushroom). Ku’ahl: *wuiy tapsh* (*wuiy* = stone; *utapch* = flower), *wuiy ugtapch chpach* (flower that comes out of the stone) or *jam shkual* (born in the water). Paipai: *shaá* (extended), *wui tabsh*, *wui tab* or *iwuil taosh* (stone flower), and *wui shishai* (lying on the stone or stone rug).

These three *Xanthoparmelia* species are used to disintegrate gallstones (*wui wui bjá* in Paipai) and relieve stomach pain (*iwuai rab* in Paipai or *tu urrap kieu* in Ku’ahl). This last condition occurs when food “*cae mal* o *pesada*” (settles bad or heavy); it can be due to an infection from eating spoiled food or stew outside the common diet. In addition, a woman mentioned that older adults from Ejido Kiliwas taught her that these lichens have an antihypertensive effect; in that location, a traditional healer described that the mosses called *alfombra de piedra* (stone carpet) were used to control hypertension.

In La Huerta, one person reports the medicinal use of *Xanthoparmelia lineola* and *X*. *mexicana*, locally known as *wui tabsh* (stone flower), to treat cancer administered as a decoction.

In Ejido Puerta Trampa, Bertha Hernández García^ꝉ^ mentions the medicinal use of *flor de piedra* (*Xanthoparmelia* spp.) to alleviate gastrointestinal disorders, such as stomach pain and *empacho*. It is believed that during *empacho* the food sticks to the stomach and inflames it; and it is caused by excessive food intake, ingesting spoiled or unwanted food, and feeling embarrassed or disgusted while eating. She explains that the lichen “nutrients” remove these foods from the stomach.

In this site, a Yaqui descent woman uses *Xanthoparmelia* spp. to cure urinary ailments such as *mal de orín* characterized by lumbar pain and white sand in the urine, which turns into kidney stones and causes infection and kidney failure leading to death. She says that lichens clean the urinary tract because they are “fresh” and calm the kidney “heat.”

In all the studied communities, tea is prepared in a clay or pewter pot with a fist or “three fingers” of lichen per liter of water (traditional measures are equivalent to approximately 3 to 7 g). They wrap the lichens in a cloth before immersing them in hot water or strain them before ingestion. The tea may be drunk once in mild ailments or up to three times a day during weeks of severe illness. According to local connoisseurs, these remedies have no adverse side effects for anyone, including children. However, avoiding irritating beverages and junk food during the treatment is recommended. In addition, for *empacho* they sweeten the tea with honey, and advise rubbing the abdomen and the back with it. In the Ejido Puerta Trampa, it is mentioned that to treat urinary tract ailments lime can be added to the remedy.

### Edible mushrooms

In general, in all the studied communities, edible mushrooms are considered healthy and energy-providing because they are natural and rich in vitamins, have fiber, and are more nutritious than meat.

In La Huerta, it is mentioned that the ancient Kumeyaay ate mushrooms to become strong and avoid illnesses, such as the flu. While in the Ejido Puerta Trampa, a women said that eating mushrooms (like *Volvopluteus* sp.) in broth or stews can help mitigate the effects of menopause.

Kiliwa speakers mention that mushrooms are called *s'pooy*, and recognize four classes: (1) *s'pooy megay* (good mushroom), *meltaay s'pooy* or *ijáau s'pooy*, an edible ethnotaxon growing on wood, probably *Inonotus cuticularis* whose consumption was not verified; or perhaps one or more species of *Volvariella* or related genera; (2) *s'pooy m'pool* or *hongos conitos* (little cone mushroom) growing on soil (*Coprinellus* sp.); (3) *maat s'pooy* (soil mushroom) described as a champignon (maybe *Agaricus* sp.), and (4) *s'pooy ku'looy* (bad mushroom) for any other inedible mushroom. The cap is named *m*´*pool* (hat) and the stipe is called *jaal*.

Diverse ways of preparing edible mushrooms were documented among the Kiliwa people. Cooked with beef rinds and *cebollín de monte* (possibly *Allium unifolium* Kellog.) [37]; as *espesadura* (thickening) where the mushrooms are ground in *metate* with *tukojaa* (red chili), flour, and lard; as a soup called *s'pooy tcha'* (mushroom broth) with ground coriander seeds; and finally, boiled in water, strained, and cooked on browned flour with rabbit meat.

In Santa Catarina, mushrooms are called *kook*, including edible such as *Volvariella bombycina*, while in Ejido Kiliwa, this species is called *hongo* (mushroom), *hongo de sauce* (willow mushroom), or *hongo de álamo* (poplar mushroom). Its consumption is a memory kept by only a few elders who still crave it but for whom it is difficult to collect.

In La Huerta, *V. bombycina* is called *jlhaush*, *jlaush*, *jlshau*, or *jlhaush asaw* (mushroom that is eaten), although it also receives other names such as *hongo* or *hongo de álamo* (poplar mushroom). Some people also recognized *Tricholoma sejunctum* and *T. equestre* as edible. Most of the interviewees mention that mushrooms are very tasty and compared them with abalone because of their high quality and exquisite flavor.

Locals from San Antonio Necua consume several mushrooms, such as *Volvopluteus* sp. and *Agaricus campestris*, warn if “their time is passed” they become poisonous, and their *acordión* (gills) turns black. Some people from Ejido Puerta Trampa, Ejido Kiliwas, and Cucapah el Mayor mentioned the *hongo de lata* (canned mushroom) (commercial *Agaricus bisporus*) in the free listings. On this last site, they also report a small white fungus from the plains (possibly *Agaricus* sp.).

Three Cucapá interviewed recognized *Ustilago maydis* as edible, they cooked it with pumpkin, onion, tomato, and occasionally with cheese. In the other communities, it is not consumed.

In Ejido Kiliwas and La Huerta, only putrefied specimens of *Phellinus* sp. were found, still recognized as edible by the locals, but only when immature. However, the edibility of this taxon was not confirmed, since it can be confused with other edible lignicolous polyporoids, such as *Laetiporus sulfureus*, reported for the region [38] but not found during this study.

In general, edible mushrooms are roasted on embers or *comal* with wild chives, salt, and a bit of oil; boiled in water, strained, and stewed minced with garlic, onion, tomato, chili, pepper, and beef lard; and as soups or *quesadillas*.

### Neurotropic mushrooms

Eight interviewees in the Cucapá community mentioned the psychotropic effects of *P. pistillaris*. They considered it a strong drug, so they did not use it, but pointed out that some foreigner hippies called them “mushrooms” and paid up to 40 USD for them. To consume them recreationally spores are inhaled, others say that the mushroom is smoked. On the other hand, a person who witnessed their consumption described that it is necessary to shake off the poisonous powder and lit the “little veins” (columella and capillicium remains), inhale the smoke, and after three or four minutes, in the words of the interviewee: “they began to fly, they were hallucinating, and became crazy.”

In all study sites, *hongo alucinante* (hallucinatory mushroom) was mentioned, but it was not found. Due to its substrate, it is also known as *hongo de caca de vaca* (cow poop mushroom). Local people say these umbrella-shaped mushrooms are consumed by hippies who get stoned, dance, and undress.

### Toxic and not used mushrooms

In the Yuman towns, there are mushrooms that despite not having a particular use, they do have cultural significance, so they are named, and their ecological importance is recognized. Even a Paipai woman said that all mushrooms are sacred because everything is sacred to the “*indios*” (a term they apply to themselves).

The term *agewtaj* (“that thing does not work” or “something that does not work”) is used to refer to the mushrooms in Cucapá. This is reflected in the negative attitudes and repulsion shown by the inhabitants towards these organisms. Thirteen interviewees considered that spores of *P. pistillaris* can cause damage to the skin and eyes, from hives to severe injuries, and are lethal when ingested; consequently, they fear them. In the past, this mushroom was used to scare children and make them cry.

Similarly, in Ejido Kiliwas and Santa Catarina they consider these mushrooms frightening since gasteroid spores cause blindness and are harmful in inhaled. A Paipai woman mentioned that toxic mushrooms (including gasteroids) are called *mat kok* (earth mushroom) and that she fears them. In Ejido Puerta Trampa, some people also perceive gasteroids as toxic, *hongos de polvo* (powdery mushrooms: *Astraeus hygrometricus*, *Lycoperdon* cf. *candidum*, and *P*. *tinctorius*).

In all the study sites, several locals remember when children, their parents and grandparents told them not to touch those mushrooms because they were harmful; today they tell their children the same.

In La Huerta, *hongo de tierra* (*Agaricus campestris*), *hongo blanco* (*Coprinus* sp.), *hongo negro* (*Montagnea arenaria*), and *hongo café* (*Peziza* sp.) were reported as toxic. While in San Antonio Necua years ago, some children playing kitchen collected a yellow mushroom umbrella-like shape that was ingested by a dog and died. The following genera are considered toxic on this site: *Clitocybe*, *Coprinus*, *Cyathus*, *Omphallothus*, *Tricholoma*, and *Xerocomus*. To treat mycetisms, they recommend drinking cooking oil to induce vomit and stimulate intestinal motility as soon as possible.

At all sites, most interviewees recognized from the photographic stimuli *Ustilago maydis* as toxic. We did not collect it since almost no corn is grown; besides, as soon it appeared, they removed it since it is considered a plague. In Santa Catarina, it is called *shaj* (which is useless). Kumeyaay speakers called it *chomat-michap* (*chomat* = seed, *michap* = white). In Ejido Puerta Trampa, *U*. *maydis* is known as the *hongo de maíz* (corn mushroom).

### Other uses for mushrooms

*Ustilago maydis* is used as fodder for sheep only in La Huerta.

On the other hand, in both Kumeyaay communities, children play kitchen with mushrooms. In San Antonio Necua, Ejido Kiliwas, and Ejido Puerta Trampa, people refer to the ludic use of gasteroids, pressing them to see how spores are expelled, to make whiskers, and kick them for fun to see them burst.

In San Antonio Necua, other local names for gasteroids are *kua kaak*, *kaak tañur*, or *pintura de cuervo* (crow painting) because they have seen these birds using gasteroids to paint themselves during their dances. Likewise, three people testify that the ancient Kumeyaay, women and men, painted their faces and bodies with these mushrooms during festivals or wars. They remember that 20 years ago, in the last invasion, they soaked the spores in a bit of water and painted lines on their skin with their fingers to distinguish allies from enemies during battles. Agustín Domínguez, a traditional authority, mentions that these lines symbolize “the indian’s spirit and strength.”

Furthermore, mushrooms and crows in the Kumeyaay region could have another relationship. According to some interviewees, when the crows rejoice and their flock flutters intensely, it is an omen of the rain and with it the mushrooms appear.

### Classification of lichens

The local classification of lichens is an issue that must be approached carefully since, in study sites, people have different perceptions about the nature of lichens. They conceive them as fungi, plants, plague, or ornaments, depending on the substrate, local name, or relationship with the phorophyte. Their classification is very variable within the same community, for example in Santa Catarina, some recognize lichens as fungi, as *lama* (slime), or as a tree decoration.

In La Huerta, they called the lichens *toji* (bad bud, plague); saxicolous foliose are known as *wui tabsh*, *musgo*, *lama*, or *pelito de la piedra* (stone little hair); most of them consider lichens as plants, only a person says they are fungi. In Ejido Puerta Trampa, in Yaqui lichens of the genus *Xanthoparmelia* are called *guata* or *flor de piedra* in Spanish and are considered as mushrooms because they adhere to rocks.

In the Cucapá community, an interviewee also thinks that lichens are mushrooms because they grow in the humidity of the rocks; another one says they are algae, three more classified them as a *lama* (slime), and one more as a plague.

In Ejido Kiliwas, one person argued that lichens are fungi since they grow during the rainy season. Saxicolous lichens are called *flor de piedra* (stone flower), while the bark foliose lichens are usually called *musgo* (moss), though *Letharia columbiana* is named *flor de la manzanita* (manzanita’s flower). In San Antonio Necua, the saxicolous foliose lichens are called *costra de la piedra* (stone crust), *musgo de las piedras* (stone moss), or *lama*. Considered as “flower” or “moss,” these lichens are perceived as plants in Kiliwas and Kumeyaay communities.

In Ejido Puerta Trampa, locals considered *Letharia columbiana* a parasitic plant called *zacatito verde* (little green grass). In Santa Catarina, people also perceived it as a pest; but, at the same time, as a decoration of the tree where it grows. It is called *toje*, *iwuil mushmá* (branch little root), *kook*, or *hongo de la manzanita* because it grows on *Arctostaphylos pungens* Kunth. There are several types of *toje*, an ethnotaxon which includes parasitic plants.

### Other uses for lichens

In Ejido Kiliwas, Ejido Puerta Trampa, and La Huerta, interviewees report that foreigners from Ensenada and other cities use *Letharia columbiana* and *Teloschistes chrysophthalmus* to decorate their homes and offices, either alone or together with the branch where it grows, using it to hang jewelry. They point out that it is sold by the kilogram or by branches; or as gifts that relatives collect in regions such as the Sierra Juárez. In La Huerta. *L*. *columbiana* (alone or with the phorophyte´s branch) is used to decorate schools and houses during different festivities such as Christmas. Indigenous people from the Sierra Juárez region use *L*. *vulpina* (L.) Hue for washing their hair and removing vermin [39]. However, we did not find it.

Among the Kiliwas, the decorative use of crustose lichens of the genera *Acarospora*, *Caloplaca*, *Candelariella*, and *Protoparmeliopsis*, is observed. They appreciate these lichens for their striking colors and the figures they formed on the rocks, them and use them as home decorations, placing them on plates and watering them often. Similarly, in Santa Catarina, foliose saxicolous lichens are used as ornaments known as *lujo* (luxury) to decorate houses or to adorn tables.

In Santa Catarina, two Paipai and Ku'ahl speaker men, and one Kumeyaay from La Huerta, describe that using a rock, a knife, or a wood or iron stick, they scrape the saxicolous lichens and draw signs, letters, or figures to signal the right path in the mountains, to leave clues, to locate a particular point, or to leave messages. A Kumeyaay from San Antonio Necua uses saxicolous lichens, such as *Xanthoparmelia* spp., stepping on them to prevent slipping from the rocks. While in Cucapah el Mayor, Inocencia González^ꝉ^ (recognized wise, native speaker) and her family believe that pulling hair-shaped saxicolous lichens can cause earthquakes since they are connected to the earth.

### Availability, extraction, and storage of mushrooms and lichens

An old woman, Paipai and Ku'ahl speaker, remembers that ancient nomads collected edible and medicinal resources in wicker baskets, such as pinions and edible mushrooms, *salvia* (*Salvia* spp.) for phlegm, and *flor de piedra* (*Xanthoparmelia* spp.) to relieve stomach pain.

In all study sites, Yumans know that lichens are perennials and mushrooms grow during the rainy seasons in summer and winter. In general, local experts indicate that edible and medicinal mushrooms are difficult to find, because of the severe droughts and deforestation in the region. They walk several hours to find mushrooms, which are consumed fresh, although gasteroids can be stored wrapped in cloths, paper bags, clay pots, or glass jars. If wet, the mushrooms are sun dehydrated before storing.

None of the medicinal mushrooms and lichens are commercialized. They are considered natural resources available to everybody. Although if someone collects many edible mushrooms, these can be exchanged for other products such as lard. Only in Cucapah el Mayor, the sale *of P. pistillaris* was recorded just for recreational purposes.

In all the study sites, except Cucapah el Mayor, lichens are abundant and collected in less than 20 min by local knowers. These are detached from the substrate by hand or using metal or wooden objects and stored in paper or plastic bags, cloths, and jars.

### Transmission of traditional knowledge

Most of the interviewees who know medicinal mushrooms or lichens mention they learned as children, through direct observation and oral tradition, mostly from their parents and grandparents; to a lesser extent from uncles or in-laws, and very rarely from outsiders (only one mention). They say that earlier, these organisms were used more often, but today young people are not interested in learning.

Sometimes traditional healers build new medicinal knowledge from their own experiences. For example, at showing the photograph of *U. maydis* to the traditional Kiliwa healer, she inferred that it could be applied to cure bleeding wounds, a user reported in other parts of Mexico [9].

## Discussion

Several specimens of mushrooms could not be determined to species because some were highly decomposed, parasitized, or already cooked; or several in good conditions did not agree with the species recorded in the literature, as in the case of *Chlorophyllum* sp. and *Volvopluteus* sp., the latter a new segregated genus in which new species have recently been discovered [40]. It is necessary to delve into the taxonomy of the collected specimens.

In this type of studies, trust between the informant and the researcher is a cornerstone [30]. Some persons even mention that not everybody shares information with strangers since this knowledge is considered a valuable treasure or secret. In the present work, trust was achieved and proven since some great sages who did not know about medicinal mushrooms or lichens willingly shared their knowledge on medicinal plants, for example, *calaguala* (*Pellaea truncata* Goodd.) for kidney and urine diseases; *gobernadora* (*Larrea tridentata* (DC.) Coville) to destroy gallstones, and *Salvia* spp. for the flu.

At the beginning of this study, some local experts requested economic compensation for the interview, arguing that some American anthropologists and linguists pay them up to 50 USD per session. However, in most cases, they agreed to be interviewed at no cost. Sometimes, at the end of the interview, their handicrafts were bought. Paid interviews may generate imprecise information and limit the research of low-income students. Following the request of several interviewees, elaboration and distribution of accessible educational materials such as posters or brochures on biocultural topics are proposed to return to the communities the product of the research.

The medicinal uses of some mushrooms and lichens recorded in this study have been documented in other Mexican regions and in several countries of the world. Lichens used to treat urinary tract disorders were reported previously in Chihuahua [41], Oaxaca [13], and China [42]. Similarly, their use to relieve stomach disorders also has been documented in India and China [5]. The lichen's effectiveness in the treatment of these diseases may be explained, in part, by the presence of secondary metabolites such as usnic acid, which has been proven to have antibacterial, antiviral, antihistamine, analgesic, anti-inflammatory, and spasmolytic activity [43].

The use of lichens by the Kiliwas to alleviate liver conditions is the first report in the country; it may also be based on metabolites with hepatoprotective activity, as has been observed in some lichens [44].

Besides the Kiliwas, the use of lichens to treat heart conditions has been recorded in other regions of Mexico [17]. For example, *Pseudevernia consocians* (Vain.) Hale & W.L. Culb. in Nahua and mestizo communities from Tlaxcala [45], ss well as from Bolivia, Bosnia-Herzegovina, China, Spain, India, and Tibet [42, 44, 45]. These cases show a pattern in lichen uses possibly explained by their metabolites with cardioprotective, antithrombotic, antiplatelet, and anticoagulant activity, as those found in *Usnea* spp. [44, 46].

The use of gasteroids to treat skin conditions has been registered in North America [9, 16, 47], Colombia [48], Siberia [49], China [2, 50] India [51], and South Africa [50]. Extracts from mature sporomes have therapeutic properties, such as antioxidant effect and tyrosinase inhibitory activity, which may explain their use to heal wounds [52], and antibacterial activity [50]; in addition the spores can absorb water from the blood, accelerating the coagulation and healing.

The documented similarities in the uses of lichens and mushrooms are considered intercultural cognitive convergences that may have two explanations: (1) it is an ancient knowledge transmitted from generation to generation, thus reaching different regions, or (2) this knowledge has emerged at different times in diverse cultures.

Local nomenclature also denotes an intercultural cognitive convergence. For example, the nomenclatural pattern of designating lichens based on the substrate: *flor de piedra* is a common name for saxicolous lichens widely documented in other regions of Mexico [13, 41, 45, 53] and from Argentina, Chile, and Spain [42]. The same applies to names that associate lichens with skin. *Xanthoparmelia* spp. is known in Paipai as *wui mokual* (*wui* = stone; *mokual* = leather, lining, shell, or skin), a name that agrees with that reported for the Tewa of North America, who call the lichens *kuk´owà* (rock skin) [54]. Similarly, *Lobaria* spp. is known as *qingwapi* (frog skin) in Yunnan, China, and *laolongpi* (dragon skin) by the Zang Tibetans [55]. In Tehuacán-Cuicatlán, Mexico they also call *piel de los árboles* (tree skin) to corticolous foliose lichens [13].

We document for the first time two anthropocentric categories for lichens. Saxicolous lichens used by the Kumeyaay as anti-slip when jumping from one rock to another. As well as the manipulation of the lichens to make indicative marks on the rocks by the Paipai, Ku'ahl, and Kumeyaay. This last use may be considered a type of lichenography that recalls the pictograms called lichenoglyphs, such as the “Thunderbird,” rock art manifested by Algonquins from Canada [56].

Kumeyaays have deep knowledge about crows, recognizing their intelligence and relationship with mushrooms. More studies are necessary to delve into the link between these two organisms.

Their mention that the crows smoke or paint themselves with the gasteroids may have three possible explanations, considering that crows present a sophisticated social behavior and use tools and medicines [57]. First, the birds, searching for food, press the mushrooms and release the spores. Second, the crows use the spores as medicine (zoopharmacognosy). Third, because of the birds' curiosity, they play with the mushrooms, as people kick them and burst them.

The record of the neurotropic attributes of *P. pistillaris* is interesting, as well as the mention in San Antonio Necua that the crows go crazy and scream when smoking gasteroids. Although the neurotropic properties of these fungi have not been proven, it is a worthy topic to explore. Schultes and Hofmann [58] reported a hallucinogenic gasteroid species (*Lycoperdon* sp*.*) used by the Raramuris in Northern Mexico. Regarding the coprophilous mushrooms with hallucinogenic properties reported in the field by the interviewees, possibly they correspond to *Psilocybe coprophila* (Fr.) Quél., registered in the region [38], or to some related species.

The free listing technique did not reveal any medicinal mushrooms or lichens because most of them are not considered fungi in the local classification; therefore, the order and frequency of mention could not be used to estimate their cultural importance. Despite being appreciated for their benefits, some fear them. It would be pertinent to analyze their position on a mycophilia-mycophobia gradient [59] to estimate the cultural significance.

The importance of medicinal mushrooms and lichens goes beyond their use value, availability, or other elements contemplated in composite indices to estimate their cultural significance [60]. Traditional knowledge of ecology, use, and local nomenclature of a fungus can give the connoisseur the satisfaction of preserving his culture and endow him with a sense of cultural identity, increasing the importance of the taxon beyond its practical significance. Some Yuman families give a special meaning to edible and medicinal mushrooms, not only for their benefit but because these are part of the culture they struggle to maintain, thus becoming symbols of their ethnic identity.

In other regions of Mexico [61, 62] and the world [4, 63], the availability and abundance of fungal resources, local mycological knowledge construction, and its transmission are in danger due to climatic alterations, different anthropogenic impacts, and the cultural transformation of the communities caused by changes in primary activities and land use. These problems were also documented in the studied localities, where they are aggravated and have provoked an extreme decrease in numbers of Yuman speakers, putting them in imminent danger of extinction, and consequently to the culture itself since with the language disappearance, the culture dies.

In La Huerta, a person who uses medicinal gasteroids, does not remember their local name. This shows that in communities whose language is about to disappear, the use of some therapeutic resources can be preserved, even if the vernacular nomenclature has been forgotten.

Among Yumans, the practices of medicinal mushrooms and lichens only live in the memory of the elderly, and only a few adults still use them. In contrast, the use of medicinal lichens and their trade have constantly increased in other regions such as the Himalayas and China [5]. However, in all the studied communities, some families work to preserve and recover their traditions, language, and their biocultural heritage. Beyond the death pact allegedly made by some members of these cultures [22], today is observed as a life pact to continue the legacy of the Yuman cultures, as mentioned in other studies [64].

According to the traditional authority’s testimony, the hegemonic societies forced Yuman to settle, taking away their freedom to be nomads. They mention that due to the loss of their lands and mobility, they left behind their life as hunters and gatherers. Today, their space is limited, and many plants, animals, and fungi early used are inaccessible to them. Therefore, it is urgent to carry out actions that integrate scientific and traditional knowledge on Yuman mushrooms and lichens, incorporating them into the sustainable management of ecosystems, to preserve and revalue their rich mycocultural heritage.

## Conclusion

The Yuman peoples are proud of their indigenous ancestry, they are very united cultures in resistance for centuries, and they fight and work together to conserve their traditions. Within these are the knowledge, practices, and beliefs around mushrooms and lichens; although not as widely used as before, they are still part of their traditional medicine and cuisine, besides other uses, which overall give the local sages a feeling of cultural identity.

Saxicolous lichens of the genus *Xanthoparmelia* and the powdery gleba gasteroids are the most important fungal taxa for medicinal use in the studied communities. These two cases demonstrate the existence of an intercultural cognitive convergence among the Yuman peoples regarding the medicinal use of mushrooms and lichens, as well as between them and other peoples around the world, despite their cultural and environmental differences.

## Supplementary Information

**Additional file 1: Annex 1**. Collection licenses.

**Additional file 2: Annex 2**. Request letter to carry out the field work signed by the local authorities.

## Data Availability

All mushroom and lichen specimens were deposited in the Herbario Nacional (MEXU), Instituto de Biología, Universidad Nacional Autónoma de México. The datasets used and/or analyzed during the current study are available from the corresponding author on reasonable request. Collection licenses are attached in Additional file 1: Annex 1.

## Abbreviations

ISE: International Society of Ethnobiology
SOLAE: Sociedad Latinoamericana de Etnobiología

## References

[1] Montoya A, Kong A, Torres-García EA, Moreno-Fuentes Á, Garibay-Orijel R (2014). Síntesis de los métodos cuantitativos empleados en etnomicología. La Etnomicología en México Estado del Arte.

[2] Hobbs C (1996). Medicinal mushrooms: an exploration of tradition, healing and culture.

[3] Kumar K (2001). *Parmelia* spp. (lichens) in ancient medicinal plant lore of India. Econ Bot.

[4] Chang YS, Lee SS (2004). Utilisation of macrofungi species in Malaysia. Fungal Divers.

[5] Yang M, Devkota S, Wang L, Scheidegger C (2021). Ethnolichenology—the use of lichens in the Himalayas and southwestern parts of China. Diversity.

[6] Dubovoy C (1968). Conocimiento de los hongos en el México antiguo. Bol Inf Soc Mex Mic.

[7] El GG (2011). uso tradicional de los hongos sagrados: pasado y presente. Etnobiología.

[8] Dibble C, Anderson A. Florentine Codex: Book XI: Earthly Things. Vol. 13. Salt Lake City: School of American Research, University of Utah. p. 132–133.

[9] Bautista-González JA, Moreno-Fuentes Á, Moreno-Fuentes Á, Garibay-Orijel R (2014). Los hongos medicinales de México. La Etnomicología en México Estado del Arte.

[10] Hernández F. Historia de las Plantas. Libro Quinto. Obras Completas Tomo II. Historia Natural de Nueva España, Vol. I. México, D.F.: Universidad Nacional Autónoma de México; 1959. p. 269

[11] Hernández F. Historia de las Plantas. Libro Vigésimo. Obras Completas Tomo III. Historia Natural de Nueva España, Vol. II. México, D.F.: Universidad Nacional Autónoma de México; 1959. p. 215.

[12] Guzmán G (2008). Diversity and use of traditional mexican medicinal fungi: a review. Int J Med Mushrooms.

[13] Bautista-González JA. Uso, conocimiento local y cosmovisión de líquenes en la región de Tehuacán-Cuicatlán. Tesis de maestría, Universidad Nacional Autónoma de México; 2017. https://eds.p.ebscohost.com/eds/detail/detail?vid=1&sid=250c46f0-5c7c-4081-b727-95c1a7fac27a%40redis&bdata=Jmxhbmc9ZXMmc2l0ZT1lZHMtbGl2ZQ%3d%3d#AN=tes.TES01000760717&db=cat02029a.

[14] Moreno-Fuentes Á, Garibay-Orijel R. La etnomicología en México: una introducción al Estado del Arte. In: Moreno-Fuentes Á, Garibay-Orijel R, editors. Etnomicología en México Estado del Arte. México D.F.: Red de Etnoecología y Patrimonio Biocultural (CONACYT)-Universidad Autónoma del Estado de Hidalgo-Instituto de Biología UNAM-Sociedad Mexicana de Micología-Asociación Etnobiológica Mexicana A.C.-GIDEM; 2014. p. 3–4.

[15] López-Sánchez HR. Agaricomycetes de la vegetación riparia del noroeste de Ensenada Baja California, México: su uso tradicional y potencial. Tesis de Maestría, Universidad Autónoma de Baja California; 2014. https://hdl.handle.net/20.500.12930/995.

[16] Felger RS, Moser MB (1974). Seri Indian pharmacopoeia. Econ Bot.

[17] Moreno-Fuentes Á, Aguirre-Acosta E, Pérez-Ramírez L (2004). Conocimiento tradicional y científico de los hongos en el estado de Chihuahua. México Etnobiología.

[18] INALI. México. Lenguas indígenas nacionales en riesgo de desaparición: Variantes lingüísticas por grado de riesgo. Ciudad de México: Instituto Nacional de Lenguas Indígenas; 2012. p. 132.

[19] Garduño E. Yumanos: Cucapá, Kiliwa, Paipai, Kumiai. Pueblos Indígenas de México en el Siglo XXI. México: Comisión Nacional para el Desarrollo de los Pueblos Indígenas; 2015. p. 184.

[20] Fernández MAS, Rojas-Berscia LM (2016). Vitalidad lingüística de la lengua paipai de Santa Catarina, Baja California. LIAMES Línguas Indígenas Am.

[21] Pastrana D. Yumanos, los indios más olvidados de México. Aristegui Noticias. 2019;1–5. https://aristeguinoticias.com/0704/mexico/yumanos-los-indios-mas-olvidados-de-mexico/.

[22] Heras A. Firman kiliwas pacto etnocida ante el desamparo del gobierno panista. La Jornada. 2006. https://www.jornada.com.mx/2006/11/21/index.php?section=estados&article=038n1est.

[23] Delgadillo-Rodríguez J (1998). Florística y Ecología del norte de Baja California.

[24] Rebman JP, Roberts NC (2012). Baja California plant field guide.

[25] INPI - INALI (Instituto Nacional de los Pueblos Indígenas - Instituto Nacional de Lenguas Indígenas). Atlas de los pueblos indígenas de México. 2018. http://atlas.inpi.gob.mx/.

[26] ISE (International Society of Ethnobiology). The ISE Code of Ethics. International Society of Ethnobiology. 2006. https://www.ethnobiology.net/.

[27] SOLAE (Sociedad Latinoamericana de Etnobiología). Código de ética para la investigación, la investigación-acción y la colaboración etnocientífica en América Latina. Etnobiología 2016;14(1):1–32.

[28] Sandoval C. Investigación cualitativa. Programa de especialización teórica, métodos y técnicas de investigación social. Bogotá: Instituto Colombiano para el Fomento de la Educación Superior; 2002. p. 311.

[29] Ruan-Soto F, García-del Valle Y, Reyes-Escutia FJ. La importancia cultural de los hongos comestibles desde las metodologías cuantitativas y cualitativas. In: Ruan-Soto F, Ramírez-Terrazo A, Montoya A, Garibay-Orijel R, editors. Métodos en etnomicología. San Cristóbal de las Casas: Instituto de Biología UNAM – Sociedad Mexicana de Micología – Grupo Interdisciplinario para el Desarrollo de la Etnomicología en México; 2020. p. 33–49.

[30] Bautista-González JA, Xolalpa-Molina S, Aguilar-Contreras A, Moreno-Fuentes Á. Construyendo una posible metodología a seguir en el estudio de los hongos medicinales de México. In: Ruan-Soto F, Ramírez-Terrazo A, Montoya A, Garibay-Orijel R, editors. Métodos en etnomicología. San Cristóbal de las Casas: Instituto de Biología UNAM – Sociedad Mexicana de Micología – Grupo Interdisciplinario para el Desarrollo de la Etnomicología en México; 2020. p. 11–32.

[31] Coker W, Couch J. The Gasteromycetes of the Eastern United States and Canada. Chapel Hill: The University of North Carolina Press; 1928. p. 283. https://ia600205.us.archive.org/17/items/gasteromycetesof00coke/gasteromycetesof00coke.pdf

[32] Esqueda M, Moreno G, Pérez-Silva E, Sánchez A, Altés A (2004). The genus *Tulostoma* in Sonora. Mexico Mycotaxon.

[33] Hernández-Navarro OE, Esqueda M, Gutiérrez A, Moreno G (2013). Species of *Disciseda* (Agaricales: Agaricaceae) in Sonora. Mexico. Rev Mex Biodivers.

[34] Hernández-Navarro E, Gutiérrez A, Vargas G, Esqueda M (2017). Nuevos registros de *Tulostoma* (Agaricales: Agaricaceae) de México. Rev Mex Biodivers.

[35] Herrera-Campos MA, Pérez-Pérez RE, Nash TH (2016). Lichens of Mexico. The Parmeliaceae-keys, distribution and specimen descriptions. Bibl Lichenol.

[36] Estrada-Ramírez A, Farldow-Espinoza L. Diccionario práctico de la lengua kiliwa. Baja California: Instituto Nacional de Lenguas Indígenas; 2004. p. 160.

[37] Moerman DE (1998). Native American ethnobotany.

[38] Ayala N, Ochoa C. Hongos conocidos de Baja California. Mexicali: Universidad Autónoma de Baca California; 1998. p. 161. https://books.google.com.mx/books?id=ev8QLIKijQgC&printsec=frontcover&source=gbs_ge_summary_r&cad=0#v=onepage&q&f=false.

[39] Palmer E. Lichens Baja California. Cambridge: Farlow Herbarium, Harvard University; 1875. p. 2. http://purl.oclc.org/net/edu.harvard.huh/guid/uuid/222b9738-7545-4afe-b186-ea676284ad2b.

[40] Montoya L, Bandala VM, Esqueda M (2021). *Volvopluteus canalipes* comb. nov. (Pluteaceae) from the Sonoran Desert of Mexico. Phytotaxa.

[41] Pennington CW (1969). The Tepehuan of Chihuahua: their material culture.

[42] Crawford SD, Ranković B (2015). Lichen used in traditional medicine. Lichen secondary metabolites bioactive properties and pharmaceutical potential.

[43] Nash TH (2008). Lichen biology.

[44] Prateeksha PBS, Bajpai R, Jadaun V, Kumar J, Kumar S (2016). The genus *Usnea*: a potent phytomedicine with multifarious ethnobotany, phytochemistry and pharmacology. RSC Adv.

[45] Montoya A, Estrada-Torres A, Caballero J (2002). Comparative ethnomycological survey of three localities from La Malinche Vulcano, Mexico. J Ethnobiol Ethnomed.

[46] Behera BC, Mahadik N, Morey M (2012). Antioxidative and cardiovascular-protective activities of metabolite usnic acid and psoromic acid produced by lichen species *Usnea complanata* under submerged fermentation. Pharm Biol.

[47] Burk WR, Fitzgerald TK (1981). Puffball usages among North American Indians. McIlvainea.

[48] Villalobos S, Mengual M (2017). Uso de los hongos, *Podaxis pistillaris*, *Inonotus rickii* y *Phellorinia herculeana* (Basidiomycetes), por la etnia wayuu en la alta guajira colombiana. Etnobiologia.

[49] Saar M (1991). Fungi in khanty folk medicine. J Ethnopharmacol.

[50] Al-Fatimi MAA, Jülich WD, Jansen R, Lindequist U (2006). Bioactive components of the traditionally used mushroom *Podaxis pistillaris*. Evid Based Complement Altern Med.

[51] Debnath S, Debnath B, Das P, Saha AK (2019). Review on an ethnomedicinal practices of wild mushrooms by the local tribes of India. J Appl Pharm Sci.

[52] Petrović P, Vunduk J, Klaus A, Carević M, Petković M, Vuković N (2019). From mycelium to spores: a whole circle of biological potency of mosaic puffball. S Afr J Bot.

[53] Mapes C, Guzmán G, Caballero J. Etnomicología purépecha: El conocimiento y uso de los hongos en la Cuenca de Pátzcuaro, Michoacán. México D. F.: Serie Etnociencia, Cuadernos de Etnobiología 2. Dirección General Culturas Populares, Sociedad Mexicana de Micología A.C., Instituto de Biología, UNAM.; 1981. p. 79.

[54] Robbins W, Harrington JP, Robbins F-M (1916). Ethnobotany of the Tewa Indians. Bur Am Ethnol Bull.

[55] Wang L, Narui T, Harada H, Culberson CF, Culberson W (2001). Ethnic uses of lichens in Yunnan. China Bryologist.

[56] Blomquist P (2011). Contextualizing the Reindeer Lake rock art.

[57] Savage C (2015). Crows: encounters with the wise guys of the avian world.

[58] Schultes RE, Hofmann A. Plantas de los dioses. Orígenes del uso de los alucinógenos. México D.F.: Fondo de Cultura Económica; 1982. p. 200.

[59] Ruan-Soto F, Caballero J, Martorell C, Cifuentes J, González-Esquinca AR, Garibay-Orijel R (2013). Evaluation of the degree of mycophilia-mycophobia among highland and lowland inhabitants from Chiapas, Mexico. J Ethnobiol Ethnomed.

[60] Garibay-Orijel R, Caballero J, Estrada-Torres A, Cifuentes J (2007). Understanding cultural significance, the edible mushrooms case. J Ethnobiol Ethnomed.

[61] Guzmán G (2008). Análisis de los estudios sobre los macromycetes de México. Rev Mex Micol.

[62] Estrada-Martínez E, Guzmán G, Cibrián-Tovar D, Ortega-Paczka R (2009). Contribución al conocimiento etnomicológico de los hongos comestibles silvestres de mercados regionales y comunidades de la Sierra Nevada (México). Interciencia.

[63] Agrahar-Murugkar D, Subbulakshmi G (2005). Nutritional value of edible wild mushrooms collected from the Khasi hills of Meghalaya. Food Chem.

[64] Caccavari-Garza E. Los kiliwas y su pacto de vida. Identidad, territorio y resistencia de un grupo yumano. Tesis de maestría, Universidad Nacional Autónoma de México, México D.F.; 2012. https://eds.s.ebscohost.com/eds/detail/detail?vid=1&sid=6b43d41c-6a9d-4194-9228-f6928c5cc556%40redis&bdata=Jmxhbmc9ZXMmc2l0ZT1lZHMtbGl2ZQ%3d%3d#AN=tes.TES01000680754&db=cat02029a.

