# Supplementary figures and images for: Traditional knowledge of medicinal mushrooms and lichens of Yuman peoples in Northern Mexico
Source: J Ethnobiol Ethnomed. 2022 Jul 30;18:52. doi: 10.1186/s13002-022-00550-8 (PMC9339201; doi:10.1186/s13002-022-00550-8)

**Additional file 1: Annex 1. Collection licenses.**


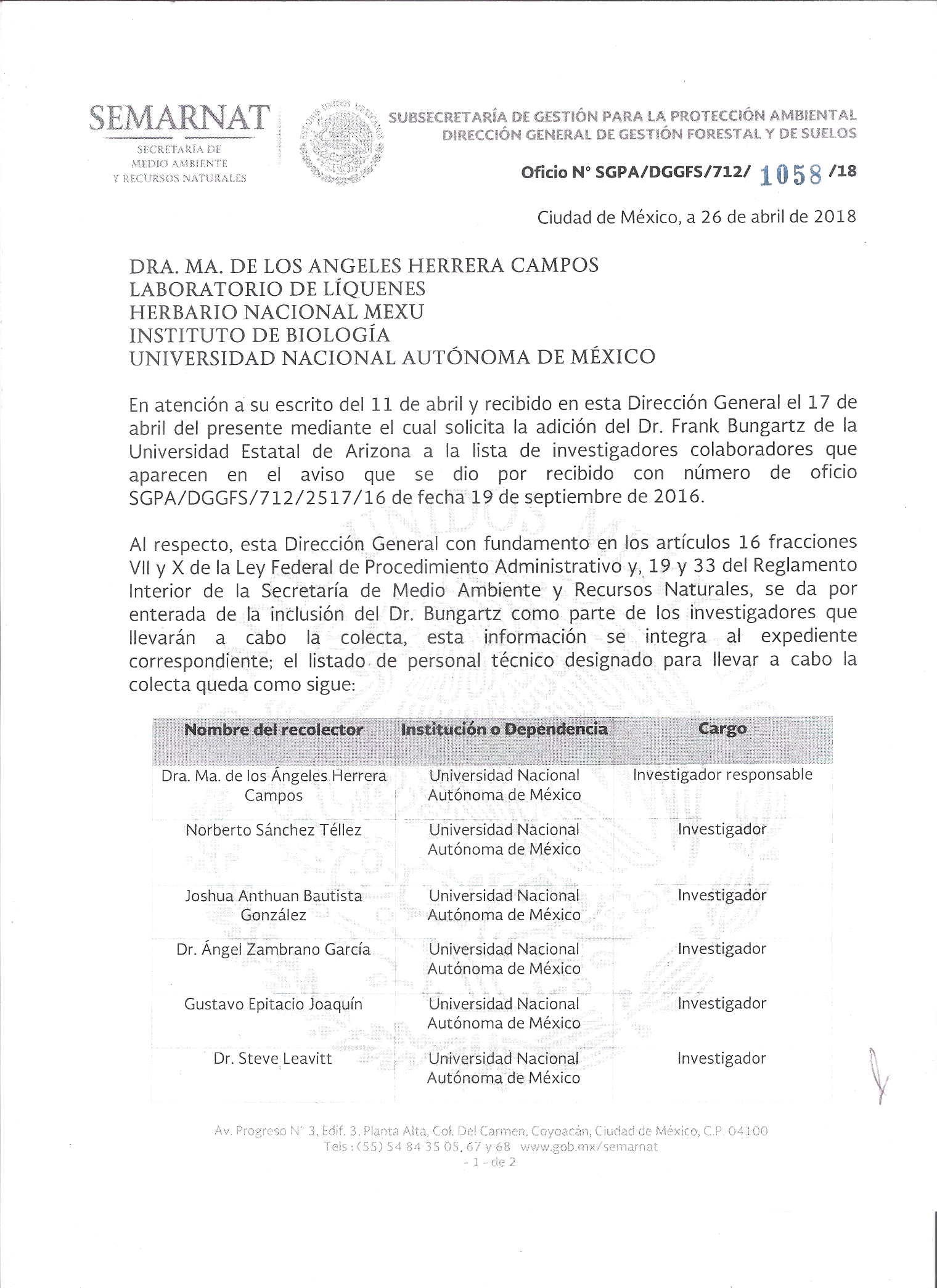


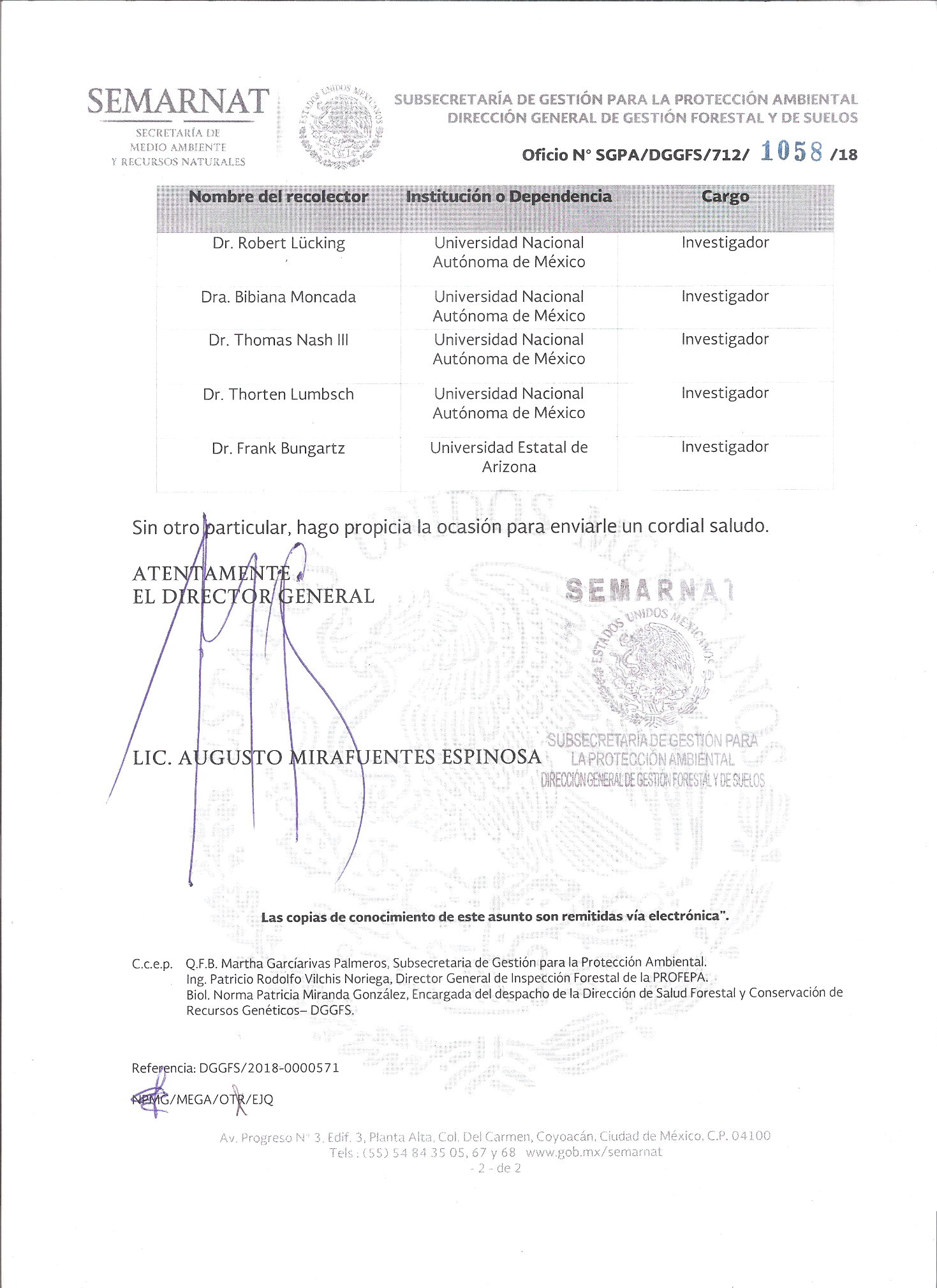


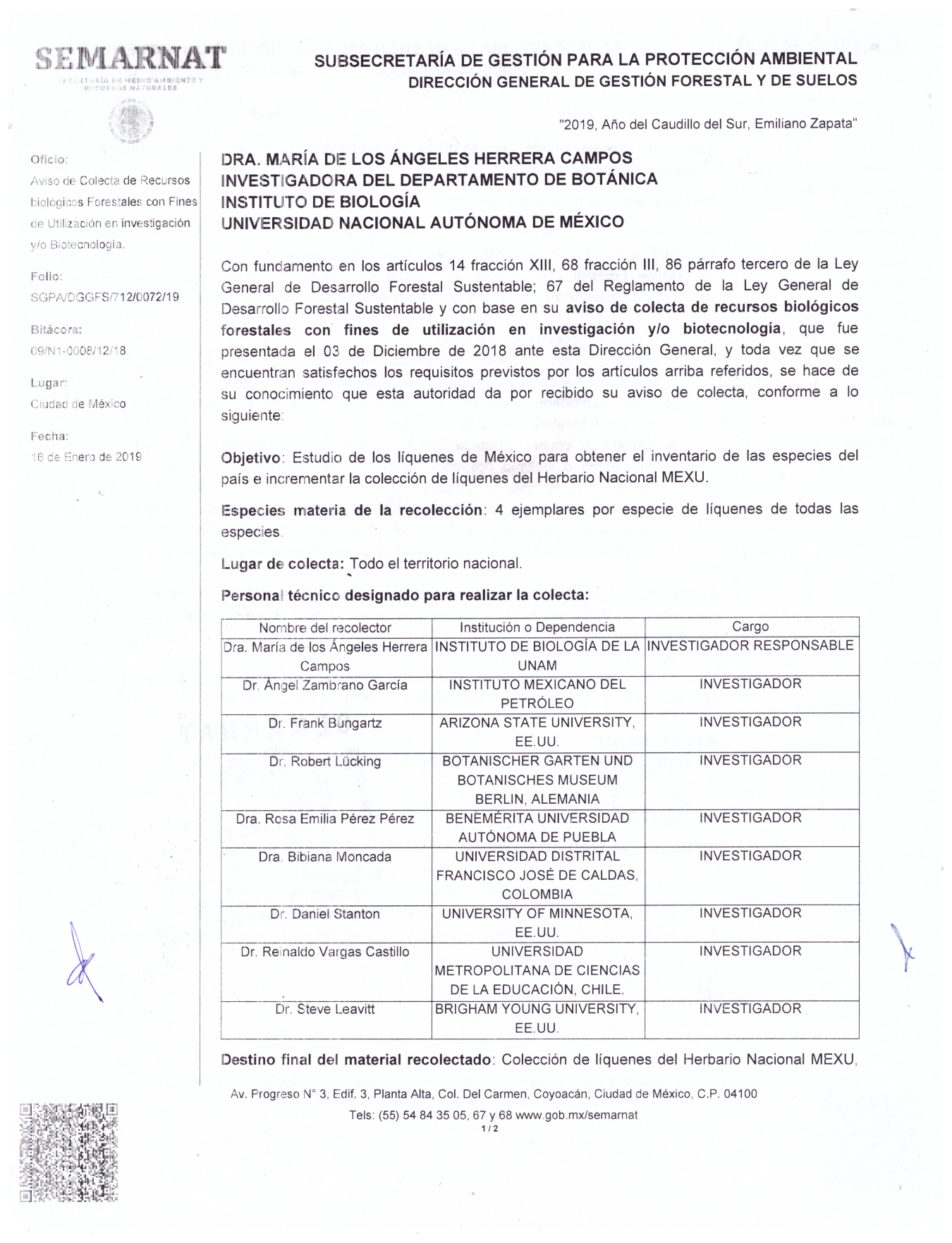


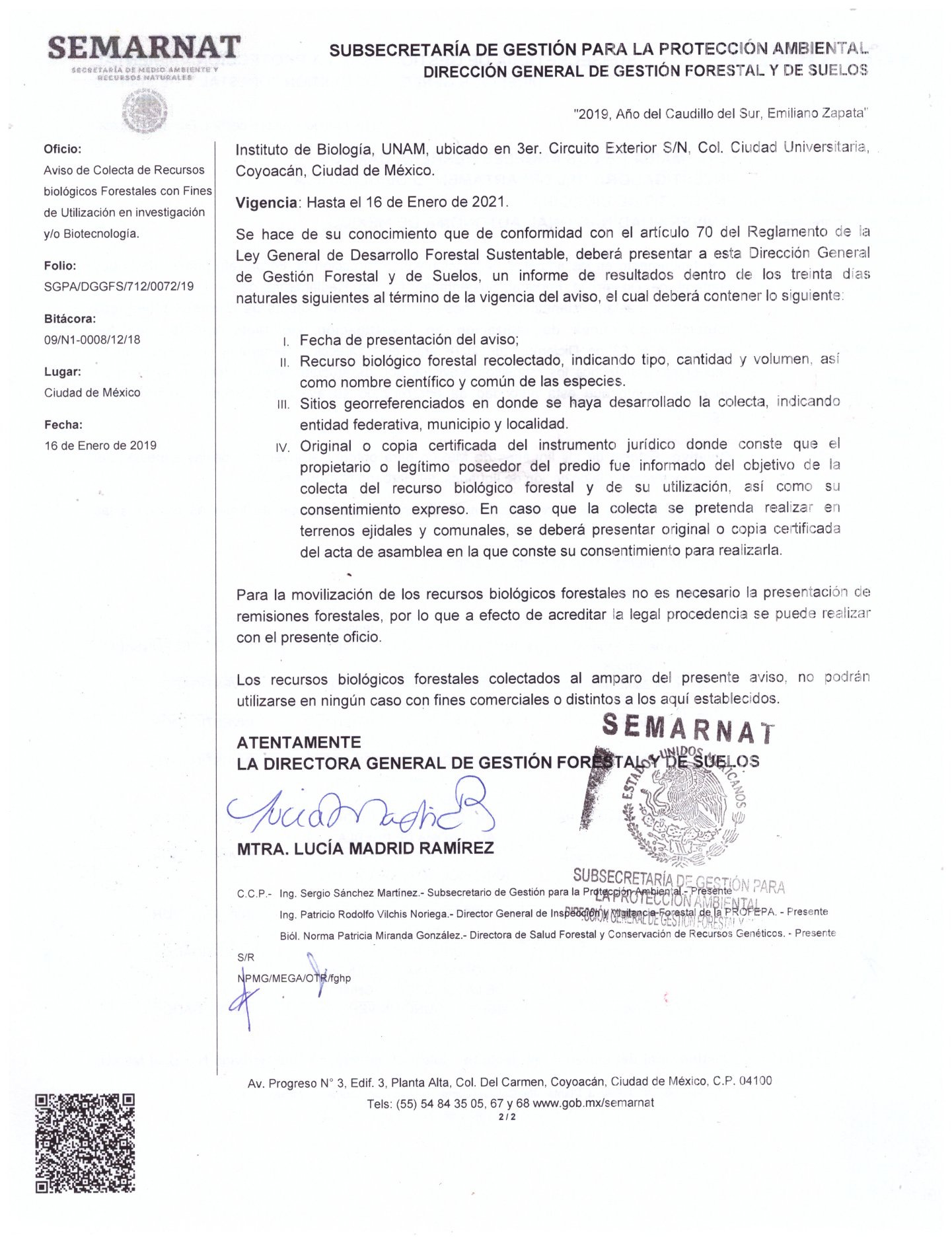

Supplement: Supplementary file 1 — Additional file 1: Annex 1. Collection licenses. [file 13002_2022_550_MOESM1_ESM.docx]
