# Supplementary material for: Traditional knowledge of medicinal mushrooms and lichens of Yuman peoples in Northern Mexico
Source: J Ethnobiol Ethnomed. 2022 Jul 30;18:52. doi: 10.1186/s13002-022-00550-8 (PMC9339201; doi:10.1186/s13002-022-00550-8)
Supplement: Supplementary file 2 — Additional file 2: Annex 2. Request letter to carry out the field work signed by the local authorities. [file 13002_2022_550_MOESM2_ESM.docx]

**Additional file 2: Annex 2. Request letter to carry out the field work signed by the local authorities.**


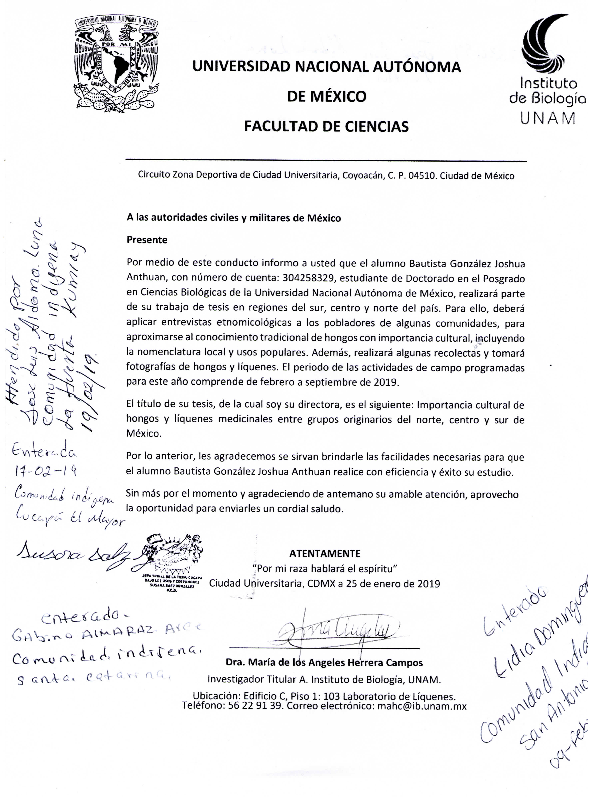


**Annex 1. Request letter to carry out the field work signed by the local authorities.**

**
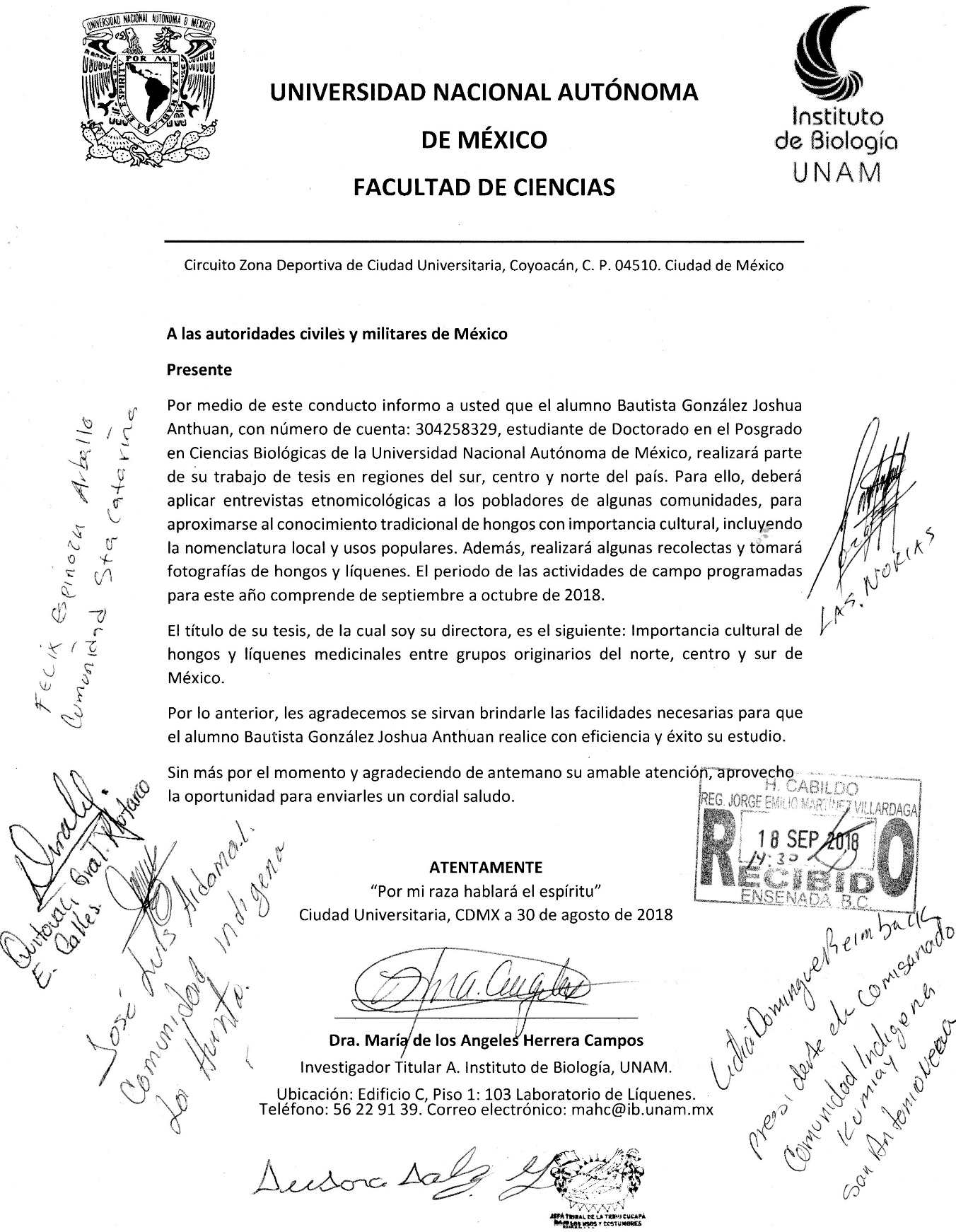
**
